# Preclinical dosimetry models and the prediction of clinical doses of novel positron emission tomography radiotracers

**DOI:** 10.1038/s41598-020-72830-w

**Published:** 2020-09-29

**Authors:** Adam A. Garrow, Jack P. M. Andrews, Zaniah N. Gonzalez, Carlos A. Corral, Christophe Portal, Timaeus E. F. Morgan, Tashfeen Walton, Ian Wilson, David E. Newby, Christophe Lucatelli, Adriana A. S. Tavares

**Affiliations:** 1 Preclinical PET-CT Facility, Edinburgh Imaging, Queen’s Medical Research Institute, The University of Edinburgh, 47 Little France Crescent, Edinburgh, EH16 4TJ UK; 2 University/BHF Centre for Cardiovascular Science, Queen’s Medical Research Institute, Edinburgh, EH16 4TJ UK; 3 Edinburgh Molecular Imaging (EMI), Nine Edinburgh Bioquarter, Edinburgh, EH16 4UX UK

**Keywords:** Biological techniques, Molecular biology

## Abstract

Dosimetry models using preclinical positron emission tomography (PET) data are commonly employed to predict the clinical radiological safety of novel radiotracers. However, unbiased clinical safety profiling remains difficult during the translational exercise from preclinical research to first-in-human studies for novel PET radiotracers. In this study, we assessed PET dosimetry data of six ^18^F-labelled radiotracers using preclinical dosimetry models, different reconstruction methods and quantified the biases of these predictions relative to measured clinical doses to ease translation of new PET radiotracers to first-in-human studies. Whole-body PET images were taken from rats over 240 min after intravenous radiotracer bolus injection. Four existing and two novel PET radiotracers were investigated: [^18^F]FDG, [^18^F]AlF-NOTA-RGDfK, [^18^F]AlF-NOTA-octreotide ([^18^F]AlF-NOTA-OC), [^18^F]AlF-NOTA-NOC, [^18^F]ENC2015 and [^18^F]ENC2018. Filtered-back projection (FBP) and iterative methods were used for reconstruction of PET data. Predicted and true clinical absorbed doses for [^18^F]FDG and [^18^F]AlF-NOTA-OC were then used to quantify bias of preclinical model predictions versus clinical measurements. Our results show that most dosimetry models were biased in their predicted clinical dosimetry compared to empirical values. Therefore, normalization of rat:human organ sizes and correction for reconstruction method biases are required to achieve higher precision of dosimetry estimates.

## Introduction

In positron emission tomography (PET) imaging, it is central to human safety that the dosimetry profile of a novel radiotracer is accurately determined from a robust preclinical model. The radiation dose is then translated to predict the amount of ionising radiation that would be experienced by human subjects prior to the radiotracer’s clinical use. This is one of several steps to achieve success for the clinical translation of new radiotracers^1^. However, the limited understanding of which preclinical model is the least biased for the prediction of novel radiotracer dosimetry profiles in first-in-human studies increases attrition in the decision-making process from preclinical to clinical translation of novel PET radiotracers.

There are several possible causes for the inconsistencies between predictive and empirical clinical dosimetry when determining the radiation safety of a newly developed PET radiotracer. The preclinical and clinical biodistribution of a given PET radiotracer may vary drastically due to a range of factors: inter-species differences in metabolic rates for the same radiotracer and differences in anatomical hierarchies in preclinical species compared to humans^2^. In addition, computer modelling software using preclinical in vivo dosimetry measurements may not account for the heterogeneous nature of organ arrangements, shapes and densities^3^.

Predictive dosimetry models that do not address one or several of these factors regularly show either significant over- or underestimation of both effective and absorbed organ dosing from PET radiotracers^4^. Differences in metabolism and anatomical barriers can be challenging to predict and minimise. Notwithstanding, it is possible to improve preclinical predictive dosimetry models if the known inaccuracies result from systematically modelling over- or underestimations, rather than unpredictable spurious causes. Mitigation of these causes could help minimise further lapses in the predictive ability of preclinical dosimetry models and improve the success rate of early-stage clinical trials for novel radiotracers.

A particularly compelling case for the development of better methods for clinical dosimetry estimates from preclinical data is PET imaging using peptide-based radiotracers. These peptides are known to often present adverse kidney kinetics, as the kidneys are commonly a primary clearing site via glomerular filtration^5^, resulting at times in prohibitive radiation dosimetry profiles for in-human translation. In the current era of theranostics^6,7^, accessible generator-based PET radionuclide production^8,9^, efficient and inexpensive ^18^F-labelling methods^10^ and an expanding arsenal of peptide PET radiotracers^11–13^, there is a need to identify rapidly and confidently lead radiotracer candidates for first-in-human translation. Therefore, the optimization of preclinical dosimetry models to improve the prediction of clinical doses of novel PET radiotracers is required.

In this study we aimed to quantify the absorbed dose from six fluorinated PET radiotracers with different biochemical properties and quantify biases introduced from distinct preclinical dosimetry models. We determined if any mathematical relationships between the biases introduced by methods for preclinical predictions of clinical dosimetry exist independently of the administered radiotracer and whether these relationships can be used to optimise future dosimetry estimates of novel PET radiotracers.

## Results

### Biodistribution and dosimetry comparison across six different PET radiotracers

**Figure 1:**
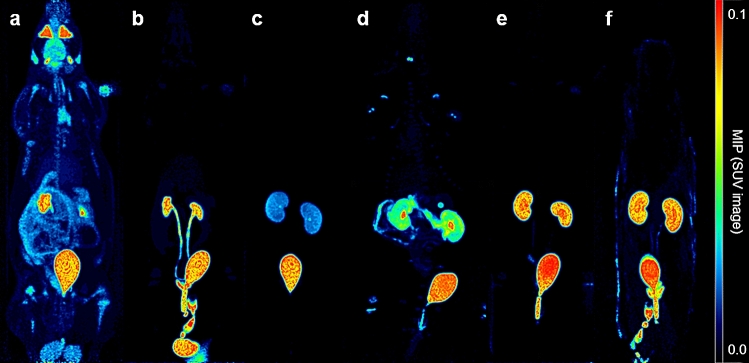
Representative Maximum Intensity Projection (MIP) of SUV PET images (0–2 g/mL) of radiotracer biodistribution in rats. Biodistribution of (**a**) [^18^F]FDG, (**b**) [^18^F]AlF-NOTA-RGDfK, (**c**) [^18^F]AlF-NOTA-NOC, (**d**) [^18^F]AlF-NOTA-OC, (**e**) [^18^F]ENC2015 and (**f**) [^18^F]ENC2018.

Representative PET images of each radiotracer biodistributions are displayed in Fig. 1 (iterative reconstruction method) and Supplementary Figure 2 (FBP reconstruction method with correspondent time-activity curves shown in Supplementary Figure 3).The highest [^18^F]FDG uptake was observed, as expected, in the brain, heart, kidneys and urinary bladder. For the peptide-based radiotracers, the highest uptake was observed in the kidneys and appeared from highest to lowest in [^18^F]ENC2015 and [^18^F]ENC2018, then [^18^F]AlF-NOTA-NOC and [^18^F]AlF-NOTA-OC and finally [^18^F]AlF-NOTA-RGDfK.

**Figure 2:**
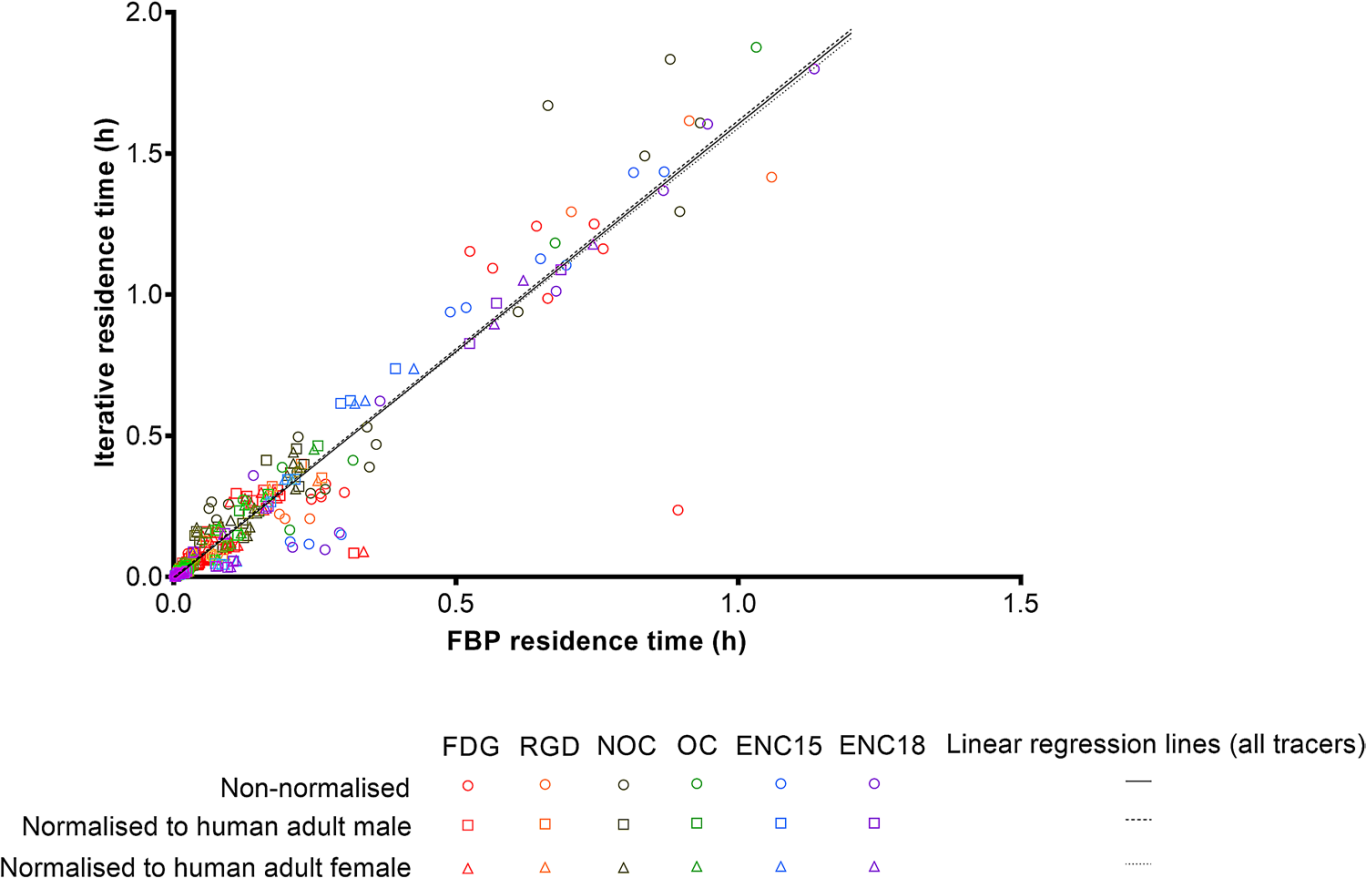
Comparison of residence times measured using FBP and iterative methods of reconstruction. Linear regression lines plotted using residence times measured for all source organ of all six radiotracers ([^18^F]FDG, [^18^F]AlF-NOTA-RGDfK, [^18^F]AlF-NOTA-NOC, [^18^F]ENC2015 and [^18^F]ENC2018).

**Figure 3:**
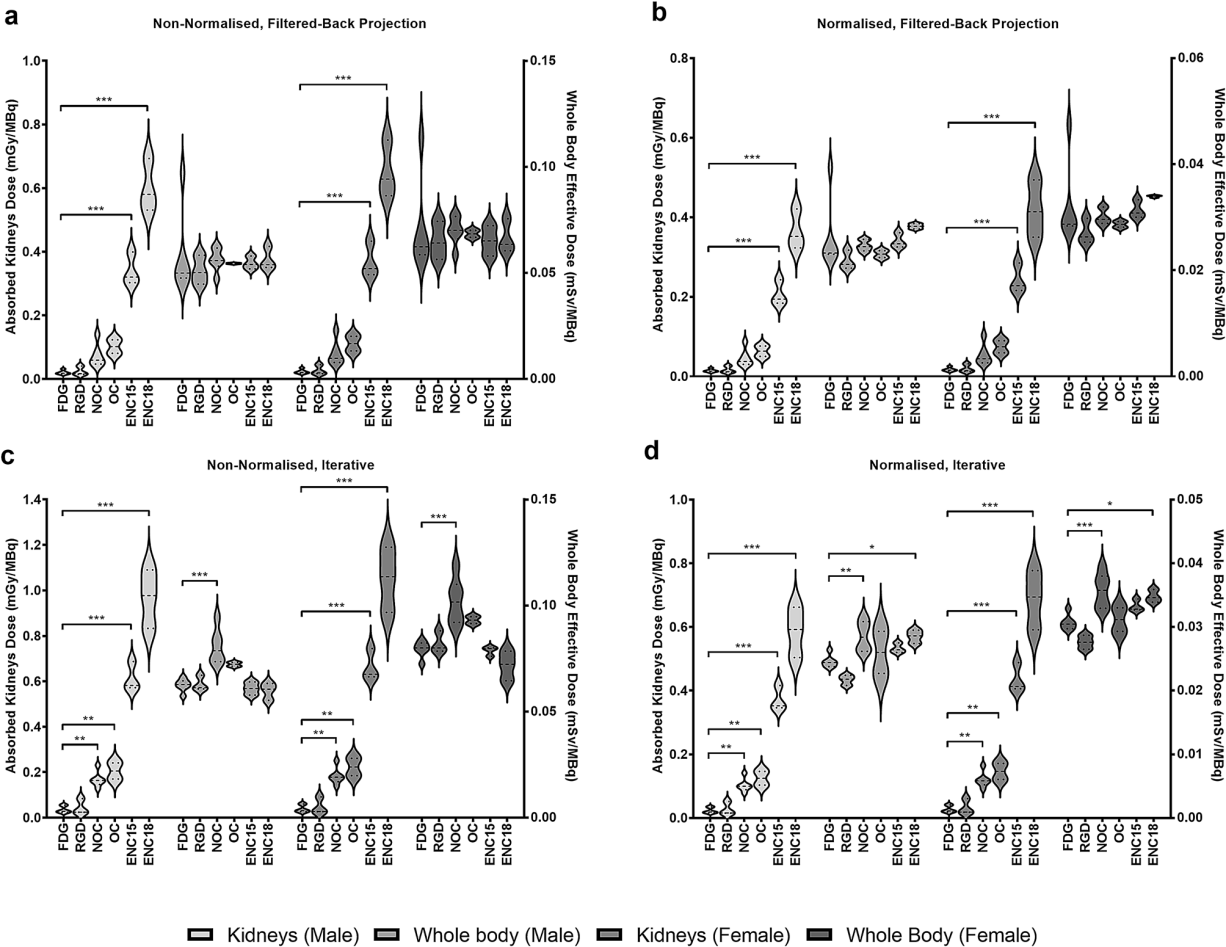
Whole-body effective doses and absorbed kidney doses, estimated using different methods of reconstruction and organ normalization strategies for all six PET radiotracers included in this study. (**a**) Doses from FBP reconstructions using non-normalised residence times, (**b**) FBP reconstructions using normalised residence times, (**c**) iterative reconstructions using non-normalised residence times and (**d**) iterative reconstructions using normalised residence times. Data presented as Mean ± SEM. One-way analysis of variants and Dunnett’s multiple comparison test ([^18^F]FDG as control group). Legend: ENC15 = [^18^F]ENC2015, ENC18 = [^18^F]ENC2018, FDG = 2-deoxy-2-[^18^F]fluoro-d-glucose, NOC = [^18^F]AlF-NOTA-NOC, OC = [^18^F]AlF-NOTA-OC, RGD = [^18^F]AlF-NOTA-RGDfK.

Residence times of radiotracers in all source organs and remaining compartment differed significantly by radiotracer except for the residence times of the intestine obtained from FBP reconstructions. All peptide-based radiotracers had significantly lower τ in the brain, heart, lungs and liver regardless of the reconstruction method used compared with [^18^F]FDG. Conversely, apart from [^18^F]AlF-NOTA-RGDfK, all peptide-based radiotracers had significantly higher τ in the kidneys compared with [^18^F]FDG. Residence times in the urinary bladder were similar for all six radiotracers used in this study (Supplementary Tables 2‒4). Organ absorbed doses and whole-body effective doses for all six PET radiotracers are summarised in Supplementary Tables 5‒12.

The dosimetry models using PET data reconstructed with FBP consistently predicted the urinary bladder, followed by the lower large intestinal (LLI) wall and kidneys, as the organs receiving the highest absorbed doses from [^18^F]FDG, independent of the phantom’s sex and normalisation of organ masses to human equivalents (Supplementary Tables 5–8). Conversely, dosimetry estimates using iterative-reconstructed PET data had more variability in the highly-dosed organs, with ranking order of LLI wall, kidneys and bladder changing depending on phantom and normalisation strategy used (Supplementary Tables 9–12). The dosimetry models yielded more consistent results for [^18^F]RGD than [^18^F]FDG; and all models predicted the top three absorbed organ doses to be in the urinary bladder > LLI wall > kidneys, except for the iterative normalised adult male model whose predicted ordering of highest absorbed organ doses was to the urinary bladder > kidneys > LLI wall. For both [^18^F]AlF-NOTA-NOC and [^18^F]AlF-NOTA-OC, all dosimetry models predicted the highest doses to be absorbed by the urinary bladder > LLI wall > kidneys, except the normalised iterative dosimetry models whose rankings were urinary bladder > kidneys > LLI wall. The predicted highest absorbed doses from [^18^F]ENC2015 were mixed depending on the dosimetry model; either the kidneys or urinary bladder were predicted to absorb the two highest doses across the models, but all models predicted the LLI wall to absorb the third-highest dose from this radiotracer. Finally, [^18^F]ENC2018 was unanimously predicted by the models to most heavily dose the kidneys, then the urinary bladder and then the LLI wall.

### Impact of PET reconstruction method and residence times’ normalisation on estimated absorbed doses

The iterative reconstruction method provided higher source organ residence time estimates than the FBP method (Fig. 2); and normalising the source organ masses of rats to male or female human equivalents did not significantly affect the ratio of the residence times estimated from FBP and iterative reconstructions (non-normalised: *r*^2^ = 0.9148; F_1,159_ = 1707; Mean = 1.611; 95% CI slope = 1.534, 1.687|normalised to adult male: *r*^2^ = 0.9101; F_1,159_ = 1609; Mean = 1.617; 95% CI slope = 1.538, 1.696|normalised to adult female: *r*^2^ = 0.9183; F_1,205_ = 1787; Mean = 1.590; 95% CI slope = 1.517, 1.664).

Given that the kidneys were predominately the main elimination route for all radiotracers and the critical organs for all six radiotracers included in this study, additional comparative analysis of predicted kidney absorbed doses was conducted alongside whole-body effective doses (Fig. 3). Data shows that both reconstruction methods and normalisation methods can have a statistically significant impact on absorbed dose estimates, but this effect is diluted when assessing whole-body effective doses. Differences across kidney absorbed doses for multiple radiotracers were less prominent when using FBP (Fig. 3a, b) compared with iterative (Fig. 3c, d) methods of reconstruction.

### Quantitative bias associated with preclinical prediction of clinical dosimetry of two PET radiotracers

**Figure 4:**
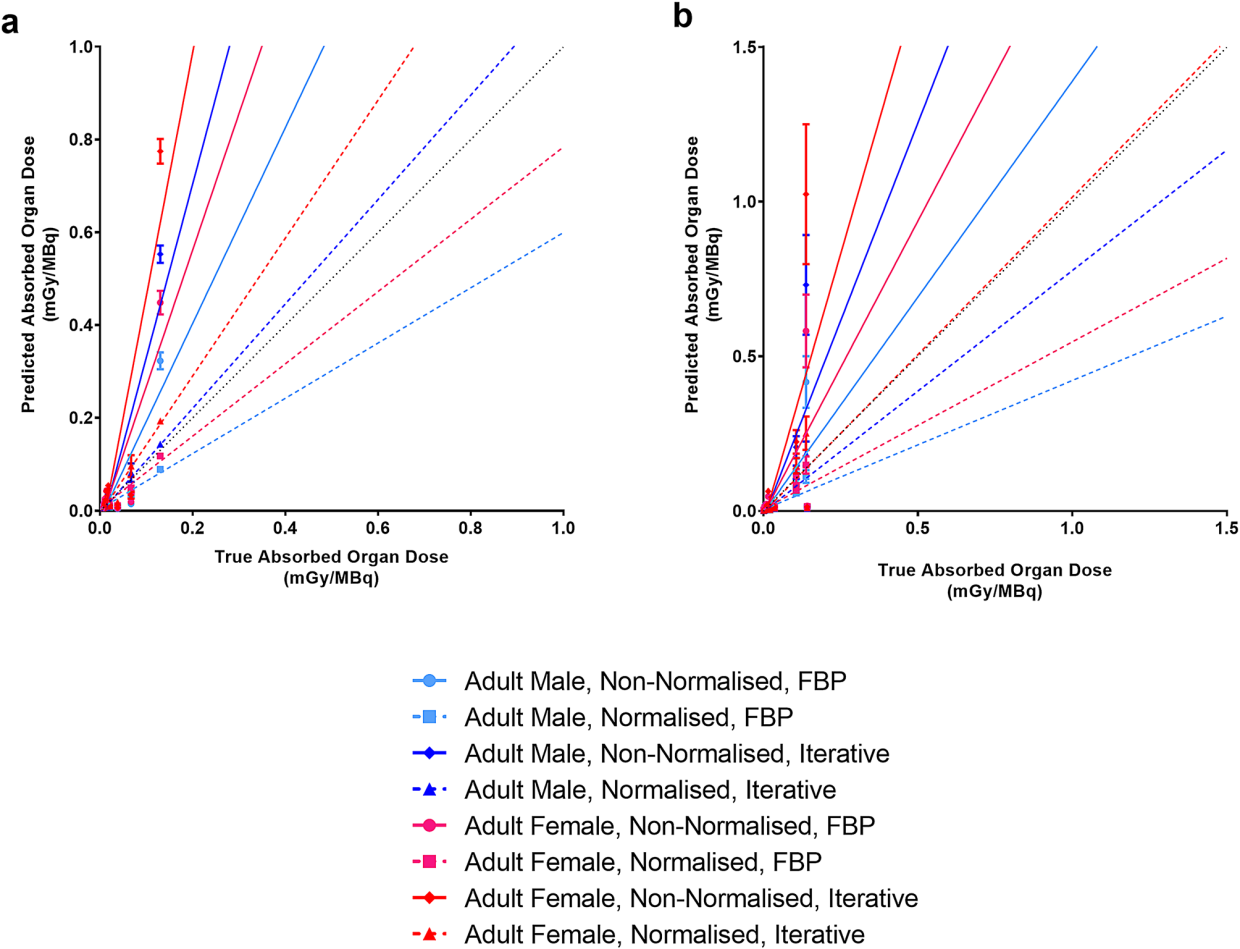
Predicted clinical absorbed organ doses from preclinical data for two PET radiotracers as a function of true measured clinical absorbed doses. Linear regressions plotted for various predictive dosimetry models of (**a**) [^18^F]FDG and (**b**) [^18^F]AlF-NOTA-OC. Resulted plotted excluding doses of the lower large intestinal wall. Data presented as Mean ± SEM. Legend: CI = Confidence interval, FBP = Filtered-back projection.

**Figure 5:**
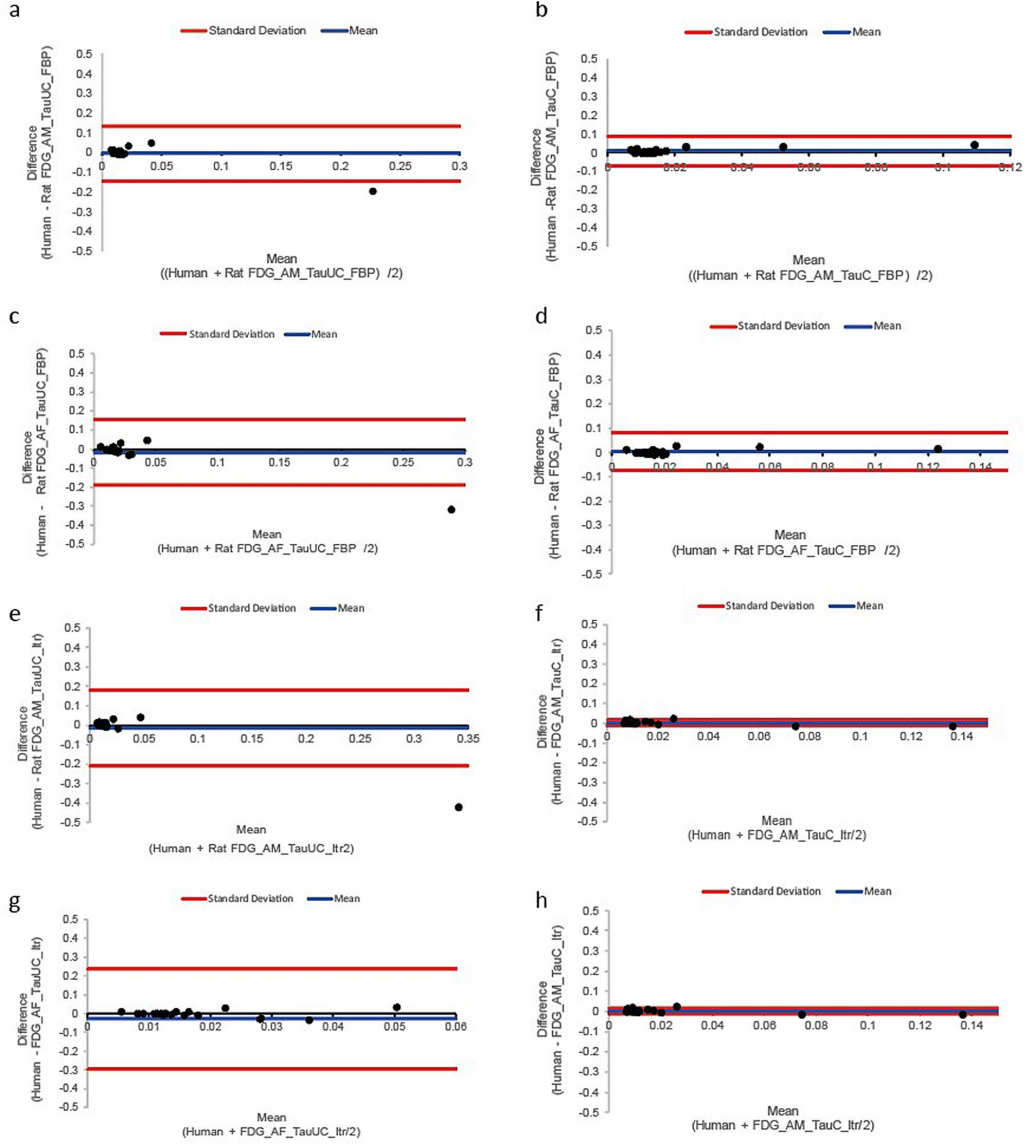
Bland–Altman plots assessing agreement between predicted clinical absorbed organ doses predicted from preclinical data for [^18^F]FDG in all tissues excluding LLI. (**a**) Human predicted dose and FDG rat adult male TauUC FBP, (**b**) Human predicted dose and FDG rat adult male TauC FBP, (**c**) Human predicted dose and FDG rat adult female TauUC FBP, (**d**) Human predicted dose and FDG rat adult female TauUC FBP, (**e**) Human predicted dose and FDG rat adult male TauUC FBP Itr, (**f**) Human predicted dose and FDG rat adult male TauC FBP Itr (**g**) Human predicted dose and FDG rat adult female TauUC FBP Itr and (**h**) Human predicted dose and OC rat adult female TauC FBP Itr.

**Figure 6:**
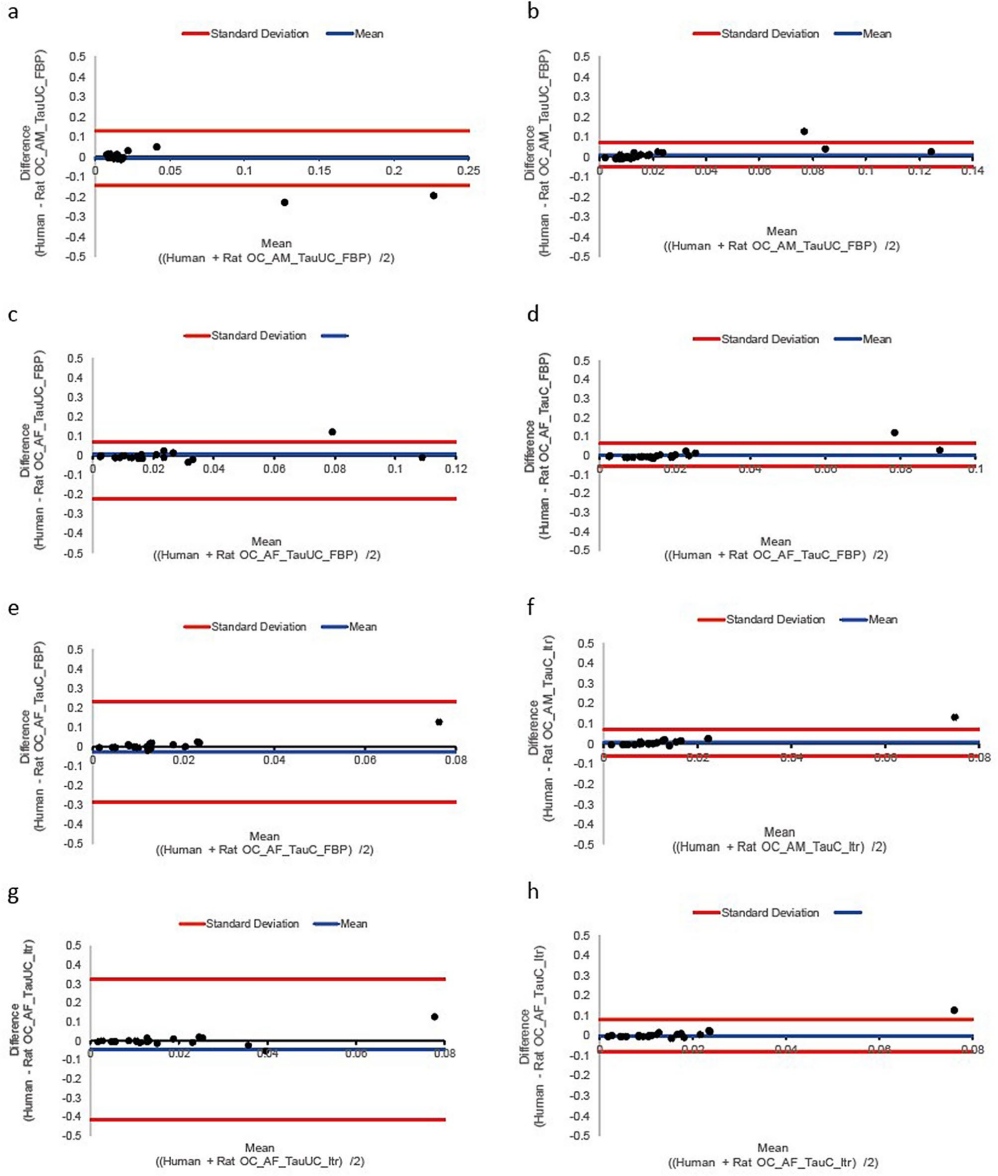
Bland–Altman plots assessing agreement between predicted clinical absorbed organ doses predicted from preclinical data for [^18^F]AlF-NOTA-NOC in all tissues excluding LLI. (**a**) Human predicted dose and OC adult male TauUC FBP, (**b**) Human predicted dose and OC rat adult male TauC FBP, (**c**) Human predicted dose and OC rat adult female TauUC FBP, (**d**) Human predicted dose and OC rat adult female TauUC FBP, (**e**) Human predicted dose and OC rat adult male TauUC FBP Itr, (**f**) Human predicted dose and OC rat adult male TauC FBP Itr (**g**) Human predicted dose and FDG rat adult female TauUC FBP Itr and (**h**) Human predicted dose and OC rat adult female TauC FBP Itr.

[^18^F]FDG bias of the preclinical dosimetry models using FBP and non-normalised τ were overestimated compared with clinically measured dosimetry values and were lower for the male than the female phantom. Conversely, absorbed doses calculated using normalised τ and FBP methods were underestimated versus clinically measured values, and these were lower for the female than the male phantom. The same bias trend was observed when using PET data reconstructed with iterative methods, except the bias was substantially amplified compared with FBP (Fig. 4a, Table 1 and Supplementary Figure 4a). Interestingly, these quantitative bias trends associated with preclinical prediction of clinical dosimetry, as a function of reconstruction method and normalised/non-normalised τ approach, measured with [^18^F]FDG (Fig. 4a, Table 1 and Supplementary Figure 4a) were identical to [^18^F]AlF-NOTA-OC (Fig. 4b, Table 1 and Supplementary Figure 4b) PET data. Bland–Altman plots assessing agreement between the two methods can be seen in Figs. 5 and 6. These show there was overall a good agreement between preclinical predictions and clinically measured doses with the kidneys as main outliers.

**Table 1:** Summary of correlation, mean bias, slope and 95% confidence intervals (CI) for [^18^F]FDG and [^18^F]AlF-NOTA-OC using different preclinical dosimetry models versus clinical measurements.

Dosimetry model	*r*^2^	Mean % bias	Slope, mean ± SEM	95% CI slope
	[^18^F]FDG			
Adult male, non-normalised, FBP	0.754	111.1	2.111 ± 0.1083	1.898, 2.323
Adult male, normalised, FBP	0.855	40.44	0.5956 ± 0.02207	0.5523, 0.6388
Adult male, non-normalised, iterative	0.786	274.2	3.742 ± 0.1752	3.398, 4.085
Adult male, normalised, iterative	0.865	11.26	1.126 ± 0.03988	1.047, 1.204
Adult female, non-normalised, FBP	0.751	194.1	2.941 ± 0.1486	2.650, 3.233
Adult female, normalised, FBP	0.846	22.07	0.7793 ± 0.02916	0.7222, 0.8365
Adult female, non-normalised, iterative	0.785	425.1	5.251 ± 0.2412	4.779, 5.724
Adult female, normalised, iterative	0.886	47.87	1.487 ± 0.04684	1.395, 1.579
	[^18^F]AlF-NOTA-OC			
Adult male, non-normalised, FBP	0.4224	39.7	1.397 ± 0.2584	0.8753, 1.920
Adult male, normalised, FBP	0.5055	58.34	0.4166 ± 0.06515	0.2849, 0.5483
Adult male, non-normalised, iterative	0.436	156	2.560 ± 0.4603	1.629, 3.490
Adult male, normalised, iterative	0.5098	22.01	0.7799 ± 0.1209	0.5356, 1.024
Adult female, non-normalised, FBP	0.3996	89.7	1.897 ± 0.3588	1.173, 2.622
Adult female, normalised, FBP	0.4917	45.96	0.5404 ± 0.08479	0.3693, 0.7116
Adult female, non-normalised, iterative	0.4119	246.7	3.467 ± 0.6393	2.177, 4.758
Adult female, normalised, iterative	0.503	1.9	1.019 ± 0.1564	0.7037, 1.335

When the LLI wall was included in the bias quantification analyses, all dosimetry models for predicting clinical absorbed organ doses of [^18^F]FDG (Supplementary Figure 4a) and [^18^F]AlF-NOTA-OC (Supplementary Figure 4b) were less precise than their respective counterparts that excluded the LLI wall from the bias quantification (Fig. 4 and Table 1). Across all tested dosimetry models, there was a range of 1.001 to 3.054 times improvement in their precision when the LLI wall was excluded from bias quantification. Bland–Altman plots assessing agreement between the two methods can be seen in Supplementary Figures 5 and 6.

## Discussion

In this study we report the dosimetry estimates for six different PET radiotracers using a preclinical rodent model. Our results showed that the choice of methods used for PET data reconstruction can impact dosimetry estimates of ^18^F-labelled radiotracers, where the τ values determined with iterative methods were approximately 50% higher than the τ values determined with FBP. Moreover, FBP OLINDA estimates were more consistent and less biased than those obtained with iterative reconstruction methods. Consequently, the use of FBP for reconstruction of preclinical PET data for dosimetry estimates is preferred.

Although the impact of reconstruction methods on radionuclide dose estimates has been sparsely investigated in previous clinical^14,15^ and preclinical^16^ studies, there is little understanding of the mathematical relationships of this bias and whether it is due to systematic errors or random and radiotracer-dependent differences. The results of the six radiotracers used in this study show a significant and strong linear relationship between predicted clinical dosimetry from preclinical models versus clinical models when using FBP and rat:human normalised data. Regardless of the radiotracer, there was always an underestimation of the predicted dose by a mean value of 42% (range 22–58%). Consequently, this mathematical relationship and simplified underestimation correction factor can be conveniently used to optimise future dosimetry estimates of novel PET radiotracers in a systematic manner. It is also encouraging to observe that the underestimation bias measured with ^18^F-labelled radiotracers was also previously reported by others using a radiotracer labelled with copper-64^16^. This suggests the mathematical relationships observed in this dosimetry study may persist in a broader nuclear medicine context and likely for different radioisotopes, including for example alpha and beta emitters used in radiation therapy studies, thus, representing a systematic error in PET dosimetry calculations based on preclinical models.

Normalization of rodent:human^17,18^, non-human primate:human^19–21^ and pig:human^17^ organ sizes has been previously used to estimate PET dosimetry of new radiotracers. Many studies focus on developing preclinical models of animal dosimetry (see review^22^) and well-established human PET dosimetry models^23,24^. Unfortunately, dichotomous dosimetry findings are prevalent when translating a novel radiotracer from preclinical research to clinical use, which is likely a result of an important knowledge gap of the bias between preclinical dose estimates and human doses. To circumvent this gap, the combined use of FBP, normalised organ data and application of the bias correction factor, as proposed in this research, represent an optimised model for translation of novel PET radiotracers to the clinic.

Results from this study also demonstrate that when the optimised dosimetry estimation model is applied, administration of [^18^F]AlF-NOTA-RGDfK will result in similar radiation dose to administration of [^18^F]FDG. The octreotide analogues would result in approximately twice the radiation dose to the kidneys and the novel radiotracers ENC2015 and ENC2018 would result in 20‒40 times higher kidney dose compared with [^18^F]FDG. The use of two octreotide analogues and two factor XIIIa analogue radiotracers also highlight the importance of carefully assessing dosimetry estimates for each radiotracer analogue prior to translation to humans, even if only small molecular structure changes are introduced.

Results from this work have direct real-world applications by setting an approach to reconstruct PET data and calculate acceptable safe dosimetry confidence intervals when translating novel PET radiotracers from animal research to clinical use, thus enabling efficient de-prioritization of radiotracers with suboptimal dosimetry values.

Although preclinical species are required for the early development of novel PET radiotracers and they can be used as models for estimation of human dosimetry, anatomical differences can be hard to circumvent. However, these differences can be minimised. For example, we observed increased precision across all dosimetry models with removal of the LLI wall from the bias quantifications. For this study’s Sprague–Dawley rat model, this modification universally improved the precision of dosimetry models. This is likely due to the fact that, unlike humans, rats have no gallbladder^25^. Consequently, it is important to take into consideration species’ anatomical differences when assessing dosimetry of novel PET radiotracers.

In conclusion, the combined use of FBP reconstruction methods normalised to rodent:human organ data and the application of bias correction factor, as proposed in this study, represent an optimised model for translation of novel PET radiotracers to the clinic and can reduce attrition when developing novel PET radiotracers.

## Materials and methods

### Radiotracer preparation

No-carrier added aqueous [^18^F]fluoride was produced via the ^18^O(p,n)^18^F nuclear reaction by irradiation of oxygen-18 enriched water on a GE PETtrace8 cyclotron. Analytical HPLC was performed on a Dionex UltiMate 300 using an Agilent Pursuit XRs 5 μm C_18_ column (250 × 4 mm). All precursors were purchased from Advanced Biochemical Compounds (ABX), apart from ENC2015 and ENC2018, which were manufactured as described previously^26^. The structures of all radiotracers prepared in this study are shown in Supplementary Figure 1. 2-Deoxy-2-[^18^F]fluoro-d-glucose was prepared using the ABX Reagents Kit for the Synthesis Module GE TRACERlab MX FDG (ABX Advanced Biochemical Compounds Ltd.).

The radiosynthesis of the Al^18^F-labelled peptides was carried out using an automated synthesis on a GE TRACERlab MX synthesiser. A disposable kit was assembled to allow the peptide conjugate to chelate aluminium fluoride and undergo purification using solid-phase extraction (SPE). Aqueous [^18^F]fluoride (15‒25 GBq) was trapped on a SepPak QMA Light cartridge (Waters) and eluted with saline (0.30 mL). This was added to a reaction vessel charged with NOTA-peptide (0.012 mL, 2.0 mM solution in 0.1 M NaOAc buffer pH 4.0) and aluminium(III) chloride (0.0060 mL, 2.0 mM solution in 0.1 M NaOAc buffer pH 4.0) in acetonitrile (0.40 mL). The reaction mixture was then heated to 100 °C for 10 min. The reaction was diluted with water (25 mL) and transferred to a SepPak C_18_ Plus Light cartridge, where it was washed with water (20 mL). The product was eluted with ethanol (1.5 mL), then water (5.5 mL) and diluted with saline (8.0 mL). [^18^F]AlF-NOTA-octreotide was obtained in 1.4 ± 0.3% radiochemical yield (starting from 23 ± 9 GBq of activity, n = 4) with a radiochemical purity of > 99%. [^18^F]AlF-NOTA-NOC was obtained in 6.4 ± 0.6% radiochemical yield (starting from 19 ± 7 GBq of activity, n = 3) with a radiochemical purity of > 99%. [^18^F]AlF-NOTA-RGDfK was obtained in 11 ± 2% radiochemical yield (starting from 21 ± 2 GBq of [^18^F]fluoride, n = 18) with a radiochemical purity of > 99%. [^18^F]ENC2015 radiochemical yield and purity were as previously reported^26^. [^18^F]ENC2018 was obtained in 10 ± 4% radiochemical yield (starting from 17 ± 0.4 GBq of [^18^F]fluoride, n = 3) with a radiochemical purity of > 99%.

### Animals

All experimental protocols were approved by an Animal Welfare and Ethical Review Body (AWERB) operating at the University of Edinburgh. The use of animals in this study was compliant with the University of Edinburgh’s institutional regulations and the Home Office’s guidance (Scientific Procedures) Act 1986. Eighteen healthy adult male Sprague–Dawley rats (443.09 ± 83.06 g, mean body weight ± SD) were used in this study. All animals were maintained and housed at the Edinburgh Preclinical Imaging facility, University of Edinburgh, UK under standard 12 h light:12 h dark conditions with food and water available ad libitum.

### PET studies

#### Image acquisition and reconstruction

On the day of imaging, rats were anaesthetised with 2‒2.5% isoflurane (50/50 oxygen/nitrous oxide, 1 L/min), then transferred to the preclinical PET/CT scanner (nanoPET/CT, Mediso, Hungary) and placed in a supine position. A CT scan (semi-circular full trajectory, maximum field of view, 480 projections, 50 kVp, 300 ms and 1:4 binning) was acquired for attenuation correction. Animals were injected intravenously in the lateral tail vein with a bolus of one of six possible radiotracers (mean ± SD): [^18^F]FDG 16.7 ± 5.53 MBq (0.3‒0.7 mL, n = 3), [^18^F]AlF-NOTA-RGDfK 25.16 ± 3.26 MBq (0.2‒0.3 mL, n = 3), [^18^F]AlF-NOTA-NOC 15.65 ± 3.80 MBq (0.2‒0.5 mL, n = 3), [^18^F]AlF-NOTA-OC 16.48 ± 5.32 MBq (0.2‒0.3 mL, n = 3), ENC2015 17.37 ± 2.10 MBq (0.1‒0.5 mL, n = 3) and ENC2018 16.49 ± 4.41 MBq (0.3‒0.4 mL, n = 3).

Immediately after radiotracer administration, a 240 min emission scan using 4 beds (50% overlap) was obtained using 3-dimensional 1:5 mode and re-binned as follows: 7 × 1 min, 2 × 5 min and 4 × 10 min. Throughout the PET/CT scanning session, animal temperature and respiration rate were monitored and controlled.

PET studies were reconstructed using filtered-back projection (FBP) and Mediso’s iterative Tera-Tomo 3D reconstruction algorithm with the following settings: 4 iterations, 6 subsets, full detector model, normal regularization, spike filter on, voxel size 0.4 mm and 400‒600 keV energy window. PET data were corrected for randoms, scatter and attenuation.

#### Image processing

Reconstructed whole-body PET/CT images were imported into PMOD 3.8 software (PMOD Technologies, Switzerland) and static regions-of-interest (ROIs) were selected in approximately every third axial slice of source organs in animal subjects, which included the kidneys (left and right), urinary bladder, lungs (right and left), heart, brain, liver and intestine. Then, PMOD’s contours interpolation tool was used to interpolate ROIs for each organ into a corresponding volume-of-interest (VOI). A static cuboidal VOI was also drawn to include the animal whole-body.

#### Dosimetry analysis

Time-activity curves for each source organ, whole-body and remainder of the body were plotted. From 240 min to infinity min, only the physical half-life of fluorine-18 was used to estimate activity. Residence times (τ) were determined for each radiotracer in source organs, whole-body and remaining (any activity assigned to the whole-body but not assigned to a source organ). Each preclinical source organ’s τ was determined by calculating the area under its respective time-activity curve (activity normalised to the percentage maximum injected dose as a function of time) using the trapezoid method. Normalised τ of each radiotracer were obtained from the product of a compartment’s preclinical τ and a scaling factor (Supplementary Table 1), the latter of which was calculated using (b_r_/o_r_)*(o_h_/b_h_) (adapted from^19^), where b_r_ and b_h_ were the body masses determined for rats and humans respectively; o_r_ and o_h_ were the individual organ masses determined for rats and humans respectively. The b_r_ used was 332.67 g (mean of^27–29^) and the b_h_ used was 73,000 g for the adult male and 60,000 g for the adult female^30^. Both measured (non-normalised) and normalised τ were applied as kinetic data to human adult male and female phantoms from OLINDA/EXM 1.0 software (Vanderbilt University, Tennessee, USA); and OLINDA/EXM subsequently provided an output of the predicted absorbed organ doses and whole-body effective doses for each phantom. From each individual animal, eight dosimetry models were constructed from all possible combinations of image reconstruction method (FBP or iterative), τ (non-normalised or normalised to human organ masses), and sex of phantom (male or female).

#### Preclinical versus clinical bias analysis

Normalised and non-normalised τ for all six tested radiotracers in different organ compartments were plotted in a scatterplot of τ from iterative reconstructions as a function of those obtained from FBP reconstructions. Regression line slopes of FBP τ as a function of iterative τ were compared with a line-of-identity (slope = 1, i.e. perfect prediction of absorbed organ doses) by determining their 95% confidence intervals to assess if the prediction of residence times differed by reconstruction method. Additionally, scatterplots of preclinically-predicted clinical absorbed organ doses from a given radiotracer as a function of the corresponding true clinical absorbed organ doses previously published per radiotracer were plotted for [^18^F]FDG^31^ and [^18^F]AlF-NOTA-OC^32^. A regression line was plotted for each radiotracer and each model’s mean percentage bias was calculated by defining the absolute value of 1 minus the mean slope of the model, multiplied by 100.

#### Graphical and statistical analysis

All graphs and statistical analyses were generated and performed in GraphPad Prism 5.01 (GraphPad Software Inc., California, USA). One-way ANOVA followed by a Dunnett’s multiple comparison post-hoc test with [^18^F]FDG as the control was used to determine which novel radiotracers’ biodistribution and dosimetry differed from [^18^F]FDG in the same organs. All statistical tests used *α* = 0.05 as the measure for statistical significance. Bland–Altman plots were calculated as previously described^33,34^.

## Supplementary information

Supplementary file1.
