# Supplementary material for: Preclinical dosimetry models and the prediction of clinical doses of novel positron emission tomography radiotracers
Source: Sci Rep. 2020 Sep 29;10:15985. doi: 10.1038/s41598-020-72830-w (PMC7525662; doi:10.1038/s41598-020-72830-w)
Supplement: Supplementary file 1 — Supplementary file1. [file 41598_2020_72830_MOESM1_ESM.docx]

**Supplementary file**

**Preclinical dosimetry models and the prediction of clinical doses of novel Positron Emission Tomography radiotracers**

Adam A. Garrow^1^, Jack P. M. Andrews^2^, Zaniah N. Gonzalez^1,2^, Carlos A. Corral^1,2^, Christophe Portal^3^, Timaeus E. F. Morgan^1,2^, Tashfeen Walton^1,2^, Ian Wilson^4^, David E. Newby^1,2^, Christophe Lucatelli^1^ & Adriana A. S. Tavares^1,2*^

**Affiliation(s):**

^1^ Edinburgh Imaging, The University of Edinburgh, Queen’s Medical Research Institute, EH16 4TJ, Edinburgh, UK

^2^ University/BHF Centre for Cardiovascular Science, Queen’s Medical Research Institute, EH16 4TJ, Edinburgh, UK

^3^ Edinburgh Molecular Imaging (EMI), Edinburgh Bioquarter, Nine, EH16 4UX, Edinburgh, UK

^4^ ImaginAb, CA 90301, Inglewood, USA

***Corresponding Author:**

Adriana A. S. Tavares, PhD

Preclinical PET-CT Facility

47 Little France Crescent, Edinburgh, EH16 4TJ, United Kingdom

E-mail: [adriana.tavares@ed.ac.uk](mailto:adriana.tavares@ed.ac.uk)

Telephone number: +44(0)131 242 6803

**Supplementary figure 1.** Chemical structures of radiotracers prepared for use in the current study: NOTA-RGDfK (**1**), NOTA-octreotide (**2**), NOTA-NOC (**3**), ENC2015 (**4**), ENC2018 (**5**) and [^18^F]FDG (**6**). NOTA-Peptide tracers **1**‒**5** are shown without chelated aluminium [^18^F]fluoride.

**Supplementary table 1.** Scaling (normalisation) factors applied to residence times of PET radiotracers in male Sprague-Dawley rats to predict human adult residence times. Legend: *: Sum of the small intestine, recto sigmoid, right colon, left colon and stomach from ^1^ †: Sum of the intestine and stomach mass ^2^ o_h_: human adult female organ weight ^1^, o_r_: male rat organ weight (Average of Spector, 1961 ^3^; Tse, 1998 ^2^; Stabin et al. 2006 ^4^); WB: Whole Body.

| **Organ** | **Average o_r_ (g)** | **Adult Male** | | | **Adult Female** | |  |
| --- | --- | --- | --- | --- | --- | --- | --- |
|  |  | **o_h_ (g)** | **Scaling factor** | | **o_h_ (g)** | **Scaling factor** |  |
| Kidneys | 2.337 | 310 | 0.605 | 276 | | 0.655 | |
| Urinary Bladder | 0.920 | 50 | 0.248 | 40 | | 0.241 | |
| Lungs | 1.603 | 1200 | 3.411 | 950 | | 3.285 | |
| Heart | 1.067 | 840 | 3.589 | 620 | | 3.223 | |
| Brain | 2.126 | 1450 | 3.108 | 1300 | | 3.390 | |
| Liver | 8.530 | 1800 | 0.962 | 1400 | | 0.910 | |
| Intestine | 18.107† | 1420* | 0.357 | 1230* | | 0.377 | |
| WB Remaining | 297.977 | 65930 | 1.008 | 54184 | | 1.008 | |

d


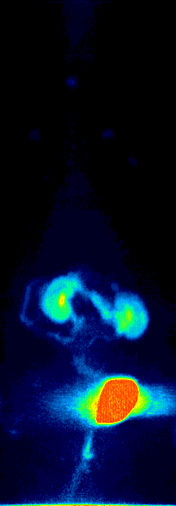

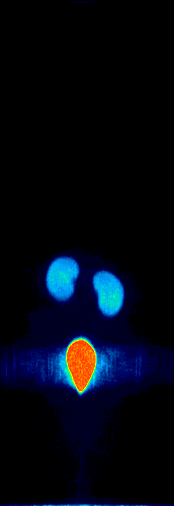

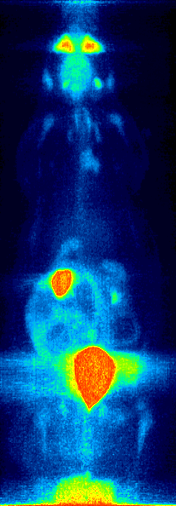

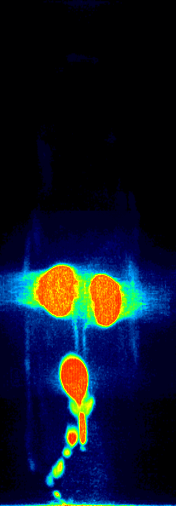

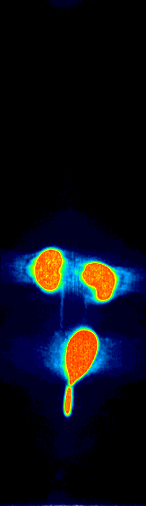

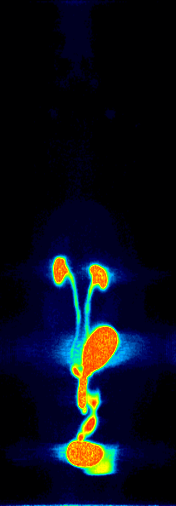

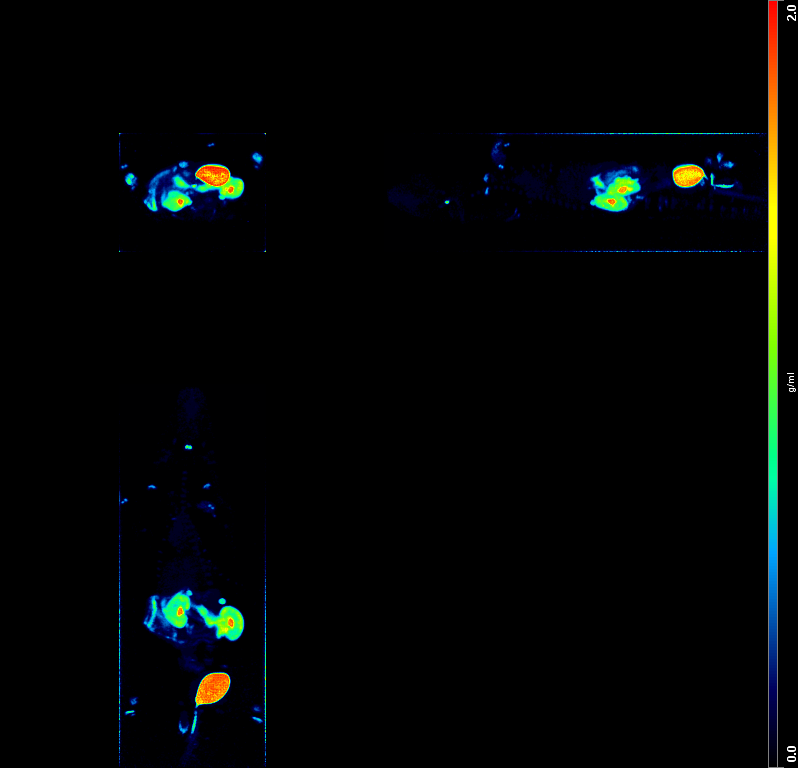

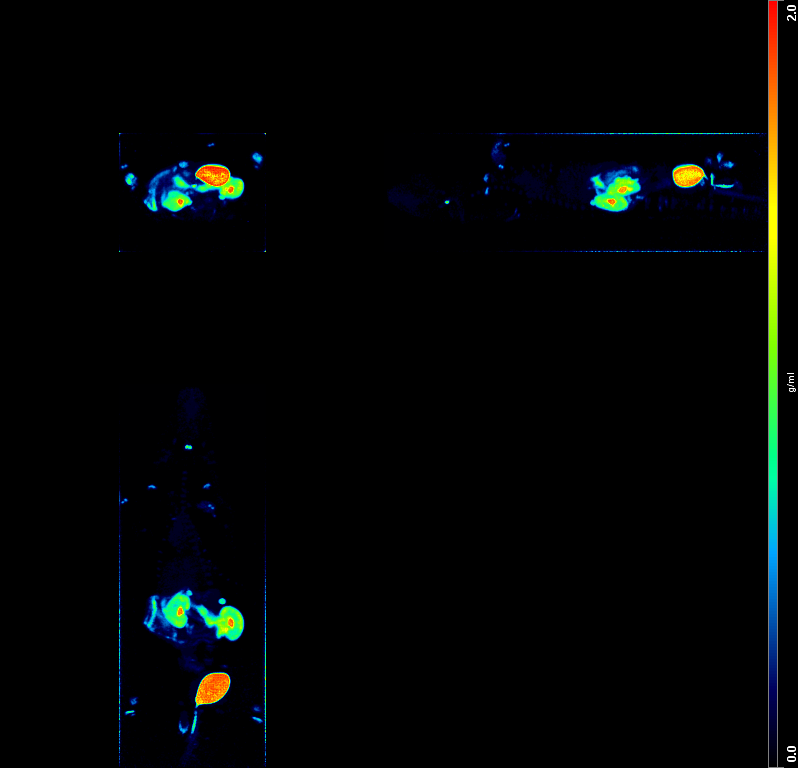


MIP

0.0

0.1

f

e

c

b

a

**Supplementary figure 2.** Representative Maximum Intensity Projection (MIP) spatial images of PET radiotracer biodistributions in male Sprague-Dawley rats from non-decay-corrected iterative reconstruction method. Legend**:** Biodistributions of 2-deoxy-2-[^18^F] fluoro-D-glucose **(a)**, [18F]NOTA-RGDfK **(b)**, [^18^F]AlF-NOTA-NOC **(c)**, [^18^F]AlF-NOTA-octreotide **(d)**, [^18^F]ENC2015 **(e)** and [^18^F]ENC2018 **(f)**. MIP: Maximum intensity projection spatial.

**Supplementary figure 3.** Time-activity curves of radiotracer uptake within identified source organs (excluding whole-body and remaining compartments) by radiotracer of male Sprague-Dawley rats. Legend: Time-activity curves are determined from filtered backprojection reconstructions for **(a)** 2-deoxy-2-[^18^F]fluoro-d-glucose, **(b)** [^18^F]AlF-NOTA-RGDfK, **(c)** [^18^F]AlF-NOTA-NOC, **(d)** [^18^F]AlF-NOTA-octreotide, **(e)** [^18^F]ENC2015 and **(f)** [^18^F]ENC2018. Time-activity curves are also determined from iterative reconstructions for these radiotracers in the same named order as filtered backprojection reconstructions **(g-l)**. Data presented as Average±SEM.

**Supplementary table 2.** Measured average residence times of PET radiotracers in male Sprague-Dawley rat source organs as determined from two reconstruction modalities.

| Organ | Residence Time (h), Average | | | | | | | | | | | |
| --- | --- | --- | --- | --- | --- | --- | --- | --- | --- | --- | --- | --- |
|  | Filtered Backprojection | | | | | | Iterative | | | | | |
|  | FDG | RGD | NOC | OC | ENC15 | ENC18 | FDG | RGD | NOC | OC | ENC15 | ENC18 |
| Brain | 1.24E-02 | 1.93E-03 | 1.59E-03 | 1.09E-03 | 9.21E-04 | 1.03E-03 | 2.54E-02 | 2.25E-03 | 2.59E-03 | 1.39E-03 | 1.10E-03 | 1.01E-03 |
| Heart | 1.58E-02 | 2.79E-03 | 3.61E-03 | 2.64E-03 | 2.15E-03 | 2.40E-03 | 3.87E-02 | 4.73E-03 | 8.52E-03 | 6.72E-03 | 3.81E-03 | 3.99E-03 |
| Intestine | 3.72E-01 | 2.09E-01 | 2.97E-01 | 2.62E-01 | 2.48E-01 | 2.58E-01 | 2.87E-01 | 2.12E-01 | 4.15E-01 | 2.91E-01 | 1.31E-01 | 1.20E-01 |
| Kidneys | 2.15E-02 | 2.90E-02 | 1.08E-01 | 1.57E-01 | 5.53E-01 | 9.82E-01 | 4.99E-02 | 6.52E-02 | 2.69E-01 | 3.31E-01 | 1.01E+00 | 1.59E+00 |
| Liver | 2.49E-02 | 2.07E-02 | 2.71E-02 | 2.11E-02 | 1.03E-02 | 1.43E-02 | 4.17E-02 | 3.91E-02 | 4.82E-02 | 3.89E-02 | 1.09E-02 | 1.38E-02 |
| Lungs | 1.55E-02 | 6.64E-03 | 8.05E-03 | 7.40E-03 | 5.20E-03 | 5.70E-03 | 1.66E-02 | 6.47E-03 | 1.11E-02 | 9.01E-03 | 4.64E-03 | 4.96E-03 |
| Urinary Bladder | 6.50E-01 | 8.92E-01 | 8.03E-01 | 8.54E-01 | 7.93E-01 | 3.95E-01 | 1.15E+00 | 1.44E+00 | 1.47E+00 | 1.53E+00 | 1.32E+00 | 6.65E-01 |
| Remaining | 2.10E+00 | 1.96E+00 | 2.03E+00 | 1.86E+00 | 1.45E+00 | 1.58E+00 | 1.35E+00 | 9.96E-01 | 8.25E-01 | 7.55E-01 | 5.07E-01 | 5.99E-01 |

**Supplementary table 3.** Normalised residence times of PET radiotracers determined using filtered backprojection reconstruction.

| Organ | Residence Time (h), Average | | | | | | | | | | | |
| --- | --- | --- | --- | --- | --- | --- | --- | --- | --- | --- | --- | --- |
|  | Filtered Backprojection | | | | | | | | | | | |
|  | Adult Male | | | | | | Adult Female | | | | | |
|  | FDG | RGD | NOC | OC | ENC15 | ENC18 | FDG | RGD | NOC | OC | ENC15 | ENC18 |
| Brain | 3.84E-02 | 6.01E-03 | 4.94E-03 | 3.38E-03 | 2.86E-03 | 3.21E-03 | 4.19E-02 | 6.56E-03 | 5.19E-03 | 3.69E-03 | 3.12E-03 | 3.50E-03 |
| Heart | 5.68E-02 | 1.00E-02 | 1.29E-02 | 9.48E-03 | 7.71E-03 | 8.62E-03 | 5.10E-02 | 8.98E-03 | 1.19E-02 | 8.51E-03 | 6.92E-03 | 7.74E-03 |
| Intestine | 1.33E-01 | 7.45E-02 | 1.06E-01 | 9.35E-02 | 8.85E-02 | 9.20E-02 | 1.40E-01 | 7.86E-02 | 1.07E-01 | 9.86E-02 | 9.33E-02 | 9.70E-02 |
| Kidneys | 1.30E-02 | 1.75E-02 | 6.55E-02 | 9.52E-02 | 3.34E-01 | 5.94E-01 | 1.41E-02 | 1.90E-02 | 7.04E-02 | 1.03E-01 | 3.62E-01 | 6.43E-01 |
| Liver | 2.39E-02 | 1.99E-02 | 2.61E-02 | 2.03E-02 | 9.89E-03 | 1.38E-02 | 2.26E-02 | 1.89E-02 | 2.51E-02 | 1.92E-02 | 9.36E-03 | 1.31E-02 |
| Lungs | 5.28E-02 | 2.27E-02 | 2.75E-02 | 2.52E-02 | 1.77E-02 | 1.94E-02 | 5.09E-02 | 2.18E-02 | 2.80E-02 | 2.43E-02 | 1.71E-02 | 1.87E-02 |
| Urinary Bladder | 1.61E-01 | 2.21E-01 | 1.99E-01 | 2.11E-01 | 1.96E-01 | 9.78E-02 | 1.57E-01 | 2.15E-01 | 2.02E-01 | 2.06E-01 | 1.91E-01 | 9.52E-02 |
| Remaining | 2.12E+00 | 1.98E+00 | 2.05E+00 | 1.87E+00 | 1.47E+00 | 1.59E+00 | 2.12E+00 | 1.98E+00 | 2.03E+00 | 1.87E+00 | 1.47E+00 | 1.59E+00 |

**Supplementary table 4.** Normalised residence times of PET radiotracers determined using iterative methods of reconstruction.

| Organ | Residence Time (h), Average | | | | | | | | | | | |
| --- | --- | --- | --- | --- | --- | --- | --- | --- | --- | --- | --- | --- |
|  | Filtered Backprojection | | | | | | Iterative | | | | | |
|  | Adult Male | | | | | | Adult Female | | | | | |
|  | FDG | RGD | NOC | OC | ENC15 | ENC18 | FDG | RGD | NOC | OC | ENC15 | ENC18 |
| Brain | 7.91E-02 | 6.98E-03 | 8.05E-03 | 4.33E-03 | 3.43E-03 | 3.13E-03 | 8.63E-02 | 7.61E-03 | 8.78E-03 | 4.72E-03 | 3.74E-03 | 3.41E-03 |
| Heart | 1.39E-01 | 1.70E-02 | 3.06E-02 | 2.41E-02 | 1.37E-02 | 1.43E-02 | 1.25E-01 | 1.52E-02 | 2.75E-02 | 2.17E-02 | 1.23E-02 | 1.29E-02 |
| Intestine | 1.02E-01 | 7.58E-02 | 1.48E-01 | 1.04E-01 | 4.67E-02 | 4.27E-02 | 1.08E-01 | 7.99E-02 | 1.56E-01 | 1.09E-01 | 4.92E-02 | 4.50E-02 |
| Kidneys | 3.01E-02 | 3.94E-02 | 1.63E-01 | 2.00E-01 | 6.09E-01 | 9.62E-01 | 3.26E-02 | 4.27E-02 | 1.76E-01 | 2.17E-01 | 6.59E-01 | 1.04E+00 |
| Liver | 4.01E-02 | 3.76E-02 | 4.64E-02 | 3.74E-02 | 1.05E-02 | 1.33E-02 | 3.80E-02 | 3.56E-02 | 4.39E-02 | 3.54E-02 | 9.92E-03 | 1.26E-02 |
| Lungs | 5.65E-02 | 2.21E-02 | 3.79E-02 | 3.07E-02 | 1.58E-02 | 1.69E-02 | 5.44E-02 | 2.12E-02 | 3.66E-02 | 2.96E-02 | 1.52E-02 | 1.63E-02 |
| Urinary Bladder | 2.84E-01 | 3.57E-01 | 3.65E-01 | 3.79E-01 | 3.28E-01 | 1.65E-01 | 2.77E-01 | 3.48E-01 | 3.55E-01 | 3.69E-01 | 3.19E-01 | 1.60E-01 |
| Remaining | 1.36E+00 | 1.00E+00 | 8.32E-01 | 7.62E-01 | 5.11E-01 | 6.04E-01 | 1.36E+00 | 1.00E+00 | 8.32E-01 | 7.61E-01 | 5.11E-01 | 6.04E-01 |

**Supplementary table 5.** Predicted clinical absorbed organ doses and whole-body effective doses of PET radiotracers using non-normalised (preclinical) adult male source organ residence times from filtered backprojection reconstructions. Legend: *: Testes data from male phantoms; †: Absorbed organ dose with highest upper bound of 95% confidence interval for dosimetry model; ‡: Absorbed organ dose with second highest upper bound of 95% confidence interval for dosimetry model; §: Absorbed organ dose with third highest upper bound of 95% confidence interval for dosimetry model.

| Target Organ | Estimated Absorbed Dose of Organ (mGy/MBq), Average | | | | | |
| --- | --- | --- | --- | --- | --- | --- |
|  | Adult Male | | | | | |
|  | FDG | RGD | NOC | OC | ENC15 | ENC18 |
| Adrenals | 1.22E-02 | 1.14E-02 | 1.34E-02 | 1.33E-02 | 1.85E-02 | 2.71E-02 |
| Brain | 4.36E-03 | 2.47E-03 | 2.48E-03 | 2.22E-03 | 1.75E-03 | 1.92E-03 |
| Breasts | 8.62E-03 | 7.90E-03 | 8.26E-03 | 7.60E-03 | 6.27E-03 | 7.07E-03 |
| Gallbladder Wall | 1.32E-02 | 1.24E-02 | 1.38E-02 | 1.33E-02 | 1.49E-02 | 1.95E-02 |
| LLI Wall | 2.39E-01† | 1.46E-01 ‡ | 1.97E-01‡ | 1.76E-01‡ | 1.65E-01§ | 1.67E-01§ |
| Small Intestine | 2.21E-02 | 1.97E-02 | 2.16E-02 | 2.06E-02 | 1.97E-02 | 2.09E-02 |
| Stomach Wall | 1.34E-02 | 1.21E-02 | 1.33E-02 | 1.25E-02 | 1.27E-02 | 1.59E-02 |
| ULI Wall | 1.76E-02 | 1.65E-02 | 1.76E-02 | 1.69E-02 | 1.63E-02 | 1.78E-02 |
| Heart Wall | 1.51E-02 | 7.37E-03 | 8.26E-03 | 7.30E-03 | 6.65E-03 | 8.03E-03 |
| Kidneys | 2.01E-02§ | 2.41E-02§ | 7.26E-02§ | 1.02E-01§ | 3.41E-01‡ | 6.01E-01† |
| Liver | 8.09E-03 | 7.25E-03 | 8.78E-03 | 8.06E-03 | 8.80E-03 | 1.26E-02 |
| Lungs | 8.07E-03 | 6.07E-03 | 6.66E-03 | 6.19E-03 | 5.52E-03 | 6.69E-03 |
| Muscle | 1.35E-02 | 1.31E-02 | 1.36E-02 | 1.30E-02 | 1.18E-02 | 1.23E-02 |
| Muscle | 1.35E-02 | 1.31E-02 | 1.36E-02 | 1.30E-02 | 1.18E-02 | 1.23E-02 |
| Testes* | 1.76E-02 | 1.85E-02 | 1.83E-02 | 1.79E-02 | 1.54E-02 | 1.25E-02 |
| Pancreas | 1.32E-02 | 1.22E-02 | 1.37E-02 | 1.34E-02 | 1.61E-02 | 2.23E-02 |
| Red Marrow | 1.32E-02 | 1.21E-02 | 1.31E-02 | 1.25E-02 | 1.23E-02 | 1.42E-02 |
| Osteogenic Cells | 1.76E-02 | 1.64E-02 | 1.71E-02 | 1.60E-02 | 1.37E-02 | 1.51E-02 |
| Skin | 9.20E-03 | 8.75E-03 | 9.11E-03 | 8.54E-03 | 7.43E-03 | 8.01E-03 |
| Spleen | 1.23E-02 | 1.15E-02 | 1.33E-02 | 1.32E-02 | 1.78E-02 | 2.56E-02 |
| Thymus | 1.07E-02 | 9.71E-03 | 1.01E-02 | 9.32E-03 | 7.59E-03 | 8.49E-03 |
| Thyroid | 1.05E-02 | 9.77E-03 | 1.01E-02 | 9.27E-03 | 7.35E-03 | 8.07E-03 |
| Urinary Bladder Wall | 3.23E-01‡ | 4.35E-01 † | 3.94E-01 † | 4.17E-01 † | 3.86E-01 † | 1.99E-01 ‡ |
| Uterus | N/A | N/A | N/A | N/A | N/A | N/A |
| Total Body | 1.38E-02 | 1.30E-02 | 1.39E-02 | 1.34E-02 | 1.32E-02 | 1.48E-02 |
| Effective Dose (mSv/MBq), Average | 5.79E-02 | 5.11E-02 | 5.63E-02 | 5.45E-02 | 5.46E-02 | 5.63E-02 |

**Supplementary table 6.** Predicted clinical absorbed organ doses and whole-body effective doses of PET radiotracers using non-normalised (preclinical) adult female source organ residence times from filtered backprojection reconstructions. Legend:*: Ovaries data from female phantoms; †: Absorbed organ dose with highest upper bound of 95% confidence interval for dosimetry model; ‡: Absorbed organ dose with second highest upper bound of 95% confidence interval for dosimetry model;§: Absorbed organ dose with third highest upper bound of 95% confidence interval for dosimetry model.

| Target Organ | Estimated Absorbed Dose of Organ (mGy/MBq), Average | | | | | | |
| --- | --- | --- | --- | --- | --- | --- | --- |
|  | Adult Female | | | | | | |
|  | FDG | RGD | NOC | OC | ENC15 | ENC18 |  |
| Adrenals | 1.56E-02 | 1.46E-02 | 1.72E-02 | 1.72E-02 | 2.43E-02 | 3.58E-02 |  |
| Brain | 5.39E-03 | 3.20E-03 | 3.23E-03 | 2.89E-03 | 2.29E-03 | 2.50E-03 |  |
| Breasts | 1.10E-02 | 1.01E-02 | 1.06E-02 | 9.73E-03 | 8.02E-03 | 8.98E-03 |  |
| Gallbladder Wall | 1.59E-02 | 1.49E-02 | 1.65E-02 | 1.58E-02 | 1.71E-02 | 2.21E-02 |  |
| LLI Wall | 2.63E-01 † | 1.62E-01 ‡ | 2.18E-01 ‡ | 1.95E-01 ‡ | 1.83E-01 § | 1.84E-01 § |  |
| Small Intestine | 2.68E-02 | 2.41E-02 | 2.64E-02 | 2.52E-02 | 2.46E-02 | 2.58E-02 |  |
| Stomach Wall | 1.66E-02 | 1.50E-02 | 1.64E-02 | 1.54E-02 | 1.52E-02 | 1.87E-02 |  |
| ULI Wall | 2.24E-02 | 2.11E-02 | 2.26E-02 | 2.16E-02 | 2.07E-02 | 2.23E-02 |  |
| Heart Wall | 1.95E-02 | 9.41E-03 | 1.05E-02 | 9.32E-03 | 8.49E-03 | 1.02E-02 |  |
| Kidneys | 2.28E-02 § | 2.70E-02 § | 7.96E-02 § | 1.11E-01 § | 3.69E-01 † | 6.52E-01 † |  |
| Liver | 1.05E-02 | 9.39E-03 | 1.13E-02 | 1.03E-02 | 1.10E-02 | 1.55E-02 |  |
| Lungs | 1.03E-02 | 7.79E-03 | 8.56E-03 | 7.98E-03 | 7.24E-03 | 8.81E-03 |  |
| Muscle | 1.67E-02 | 1.61E-02 | 1.68E-02 | 1.60E-02 | 1.46E-02 | 1.52E-02 |  |
| Ovaries* | 4.29E-02 | 3.85E-02 | 4.18E-02 | 3.99E-02 | 3.65E-02 | 3.24E-02 |  |
| Pancreas | 1.65E-02 | 1.53E-02 | 1.71E-02 | 1.66E-02 | 1.94E-02 | 2.66E-02 |  |
| Red Marrow | 1.64E-02 | 1.52E-02 | 1.64E-02 | 1.56E-02 | 1.53E-02 | 1.72E-02 |  |
| Osteogenic Cells | 2.29E-02 | 2.13E-02 | 2.24E-02 | 2.08E-02 | 1.79E-02 | 1.98E-02 |  |
| Skin | 1.14E-02 | 1.08E-02 | 1.13E-02 | 1.06E-02 | 9.12E-03 | 9.82E-03 |  |
| Spleen | 1.55E-02 | 1.45E-02 | 1.67E-02 | 1.66E-02 | 2.20E-02 | 3.16E-02 |  |
| Thymus | 1.38E-02 | 1.25E-02 | 1.31E-02 | 1.20E-02 | 9.74E-03 | 1.09E-02 |  |
| Thyroid | 1.24E-02 | 1.15E-02 | 1.19E-02 | 1.09E-02 | 8.61E-03 | 9.43E-03 |  |
| Urinary Bladder Wall | 4.48E-01 ‡ | 6.07E-01 † | 5.48E-01 † | 5.82E-01 † | 5.39E-01 ‡ | 2.75E-01 ‡ |  |
| Uterus | 4.34E-02 | 4.81E-02 | 4.71E-02 | 4.72E-02 | 4.29E-02 | 3.11E-02 |  |
| Total Body | 1.72E-02 | 1.63E-02 | 1.74E-02 | 1.67E-02 | 1.65E-02 | 1.86E-02 |  |
| Effective Dose (mSv/MBq), Average ± SD | 7.08E-02 | 6.49E-02 | 7.02E-02 | 6.85E-02 | 6.51E-02 | 6.65E-02 |  |

**Supplementary table 7.** Predicted clinical absorbed organ doses and whole-body effective doses of PET radiotracers using normalised adult male source organ residence times from filtered backprojection reconstructions. Legend: *: Testes data from male phantoms; †: Absorbed organ dose with highest upper bound of 95% confidence interval for dosimetry model; ‡: Absorbed organ dose with second highest upper bound of 95% confidence interval for dosimetry model; §: Absorbed organ dose with third highest upper bound of 95% confidence interval for dosimetry model.

| Target Organ | Estimated Absorbed Dose of Organ (mGy/MBq), Average | | | | | |
| --- | --- | --- | --- | --- | --- | --- |
|  | Adult Male | | | | | |
|  | FDG | RGD | NOC | OC | ENC15 | ENC18 |
| Adrenals | 1.23E-02 | 1.10E-02 | 1.24E-02 | 1.20E-02 | 1.42E-02 | 1.98E-02 |
| Brain | 8.73E-03 | 3.17E-03 | 3.06E-03 | 2.62E-03 | 2.09E-03 | 2.29E-03 |
| Breasts | 9.14E-03 | 8.04E-03 | 8.40E-03 | 7.69E-03 | 6.20E-03 | 6.91E-03 |
| Gallbladder Wall | 1.25E-02 | 1.15E-02 | 1.25E-02 | 1.17E-02 | 1.17E-02 | 1.51E-02 |
| LLI Wall | 9.22E- | 5.79E-02 ‡ | 7.65E-02 ‡ | 6.85E-02 § | 6.33E-02 § | 6.52E-02 § |
| Small Intestine | 1.55E-02 | 1.40E-02 | 1.51E-02 | 1.41E-02 | 1.29E-02 | 1.45E-02 |
| Stomach Wall | 1.26E-02 | 1.13E-02 | 1.21E-02 | 1.13E-02 | 1.05E-02 | 1.28E-02 |
| ULI Wall | 1.39E-02 | 1.28E-02 | 1.36E-02 | 1.27E-02 | 1.16E-02 | 1.33E-02 |
| Heart Wall | 3.82E-02 | 1.15E-02 | 1.35E-02 | 1.11E-02 | 9.33E-03 | 1.08E-02 |
| Kidneys | 1.43E-02 § | 1.64E-02 § | 4.58E-02 § | 6.33E-02 ‡ | 2.07E-01 † | 3.65E-01 † |
| Liver | 7.98E-03 | 6.81E-03 | 8.11E-03 | 7.24E-03 | 6.87E-03 | 9.49E-03 |
| Lungs | 1.48E-02 | 8.79E-03 | 9.91E-03 | 9.11E-03 | 7.25E-03 | 8.34E-03 |
| Muscle | 1.13E-02 | 1.05E-02 | 1.10E-02 | 1.02E-02 | 8.81E-03 | 9.79E-03 |
| Testes* | 1.22E-02 | 1.18E-02 | 1.21E-02 | 1.14E-02 | 9.27E-03 | 9.06E-03 |
| Pancreas | 1.31E-02 | 1.17E-02 | 1.29E-02 | 1.22E-02 | 1.29E-02 | 1.70E-02 |
| Red Marrow | 1.11E-02 | 9.94E-03 | 1.06E-02 | 9.93E-03 | 9.17E-03 | 1.08E-02 |
| Osteogenic Cells | 1.67E-02 | 1.53E-02 | 1.60E-02 | 1.47E-02 | 1.22E-02 | 1.36E-02 |
| Skin | 8.54E-03 | 7.88E-03 | 8.22E-03 | 7.59E-03 | 6.33E-03 | 7.04E-03 |
| Spleen | 1.20E-02 | 1.09E-02 | 1.22E-02 | 1.18E-02 | 1.36E-02 | 1.87E-02 |
| Thymus | 1.17E-02 | 9.99E-03 | 1.04E-02 | 9.54E-03 | 7.63E-03 | 8.45E-03 |
| Thyroid | 1.09E-02 ± 1.11E-03 | 9.88E-03 | 1.02E-02 | 9.38E-03 | 7.40E-03 | 8.09E-03 |
| Urinary Bladder Wall | 8.88E-02 ‡ | 1.16E-01 † | 1.06E-01 † | 1.11E-01 † | 1.02E-01 ‡ | 5.61E-02 ‡ |
| Uterus | N/A | N/A | N/A | N/A | N/A | N/A |
| Total Body | 1.17E-02 | 1.05E-02 | 1.12E-02 | 1.05E-02 | 9.66E-03 | 1.13E-02 |
| Effective Dose (mSv/MBq), Average ± SD | 2.62E-02 | 2.18E-02 | 2.46E-02 | 2.31E-02 | 2.55E-02 | 2.83E-02 |

**Supplementary table 8.** Predicted clinical absorbed organ doses and whole-body effective doses of PET radiotracers using normalised adult female source organ residence times from filtered backprojection reconstructions. Legend: *: Ovaries data from female phantoms; †: Absorbed organ dose with highest upper bound of 95% confidence interval for dosimetry model; ‡: Absorbed organ dose with second highest upper bound of 95% confidence interval for dosimetry model; §: Absorbed organ dose with third highest upper bound of 95% confidence interval for dosimetry model.

| Target Organ | Estimated Absorbed Dose of Organ (mGy/MBq), Average | | | | | | |
| --- | --- | --- | --- | --- | --- | --- | --- |
|  | Adult Female | | | | | | |
|  | FDG | RGD | NOC | OC | ENC15 | ENC18 |  |
| Adrenals | 1.55E-02 | 1.41E-02 | 1.59E-02 | 1.55E-02 | 1.91E-02 | 2.68E-02 |  |
| Brain | 1.10E-02 | 4.10E-03 | 3.93E-03 | 3.41E-03 | 2.72E-03 | 2.96E-03 |  |
| Breasts | 1.16E-02 | 1.02E-02 | 1.06E-02 | 9.79E-03 | 7.90E-03 | 8.81E-03 |  |
| Gallbladder Wall | 1.48E-02 | 1.36E-02 | 1.47E-02 | 1.39E-02 | 1.38E-02 | 1.75E-02 |  |
| LLI Wall | 1.07E-01 † | 6.76E-02 ‡ | 8.61E-02 ‡ | 7.97E-02 § | 7.37E-02 § | 7.47E-02 § |  |
| Small Intestine | 1.83E-02 | 1.64E-02 | 1.77E-02 | 1.68E-02 | 1.56E-02 | 1.77E-02 |  |
| Stomach Wall | 1.57E-02 | 1.41E-02 | 1.50E-02 | 1.41E-02 | 1.29E-02 | 1.56E-02 |  |
| ULI Wall | 1.75E-02 | 1.62E-02 | 1.72E-02 | 1.61E-02 | 1.47E-02 | 1.68E-02 |  |
| Heart Wall | 4.52E-02 | 1.39E-02 | 1.65E-02 | 1.35E-02 | 1.14E-02 | 1.35E-02 |  |
| Kidneys | 1.70E-02 § | 1.95E-02 § | 5.37E-02 § | 7.45E-02 ‡ | 2.43E-01 † | 4.19E-01 † |  |
| Liver | 1.01E-02 | 8.65E-03 | 1.03E-02 | 9.21E-03 | 8.80E-03 | 1.21E-02 |  |
| Lungs | 1.82E-02 | 1.10E-02 | 1.27E-02 | 1.14E-02 | 9.22E-03 | 1.07E-02 |  |
| Muscle | 1.40E-02 | 1.29E-02 | 1.35E-02 | 1.26E-02 | 1.10E-02 | 1.23E-02 |  |
| Ovaries* | 2.42E-02 | 2.14E-02 | 2.30E-02 | 2.16E-02 | 1.89E-02 | 1.91E-02 |  |
| Pancreas | 1.63E-02 | 1.47E-02 | 1.60E-02 | 1.53E-02 | 1.61E-02 | 2.10E-02 |  |
| Red Marrow | 1.37E-02 | 1.23E-02 | 1.32E-02 | 1.24E-02 | 1.14E-02 | 1.34E-02 |  |
| Osteogenic Cells | 2.19E-02 | 2.00E-02 | 2.08E-02 | 1.93E-02 | 1.60E-02 | 1.79E-02 |  |
| Skin | 1.06E-02 | 9.76E-03 | 1.02E-02 | 9.41E-03 | 7.84E-03 | 8.72E-03 |  |
| Spleen | 1.51E-02 | 1.38E-02 | 1.53E-02 | 1.49E-02 | 1.76E-02 | 2.39E-02 |  |
| Thymus | 1.48E-02 | 1.28E-02 | 1.33E-02 | 1.22E-02 | 9.77E-03 | 1.08E-02 |  |
| Thyroid | 1.27E-02 | 1.16E-02 | 1.20E-02 | 1.10E-02 | 8.66E-03 | 9.46E-03 |  |
| Urinary Bladder Wall | 1.18E-01 ‡ | 1.54E-01 † | 1.47E-01 † | 1.48E-01 † | 1.36E-01 ‡ | 7.46E-02 ‡ |  |
| Uterus | 2.28E-02 | 2.28E-02 | 2.34E-02 | 2.23E-02 | 1.93E-02 | 1.76E-02 |  |
| Total Body | 1.46E-02 | 1.31E-02 | 1.40E-02 | 1.31E-02 | 1.22E-02 | 1.44E-02 |  |
| Effective Dose (mSv/MBq), Average ± SD | 3.21E-02 | 2.71E-02 | 3.01E-02 | 2.87E-02 | 3.14E-02 | 3.39E-02 |  |

**Supplementary table 9.** Predicted clinical absorbed organ doses and whole-body effective doses of PET radiotracers using non-normalised (preclinical) adult male source organ residence times from iterative reconstructions. Legend: *: Testes data from male phantoms; †: Absorbed organ dose with highest upper bound of 95% confidence interval for dosimetry model, ‡: Absorbed organ dose with second highest upper bound of 95% confidence interval for dosimetry model, §: Absorbed organ dose with third highest upper bound of 95% confidence interval for dosimetry model.

| Target Organ | Estimated Absorbed Dose of Organ (mGy/MBq), Average ± SD | | | | | | |
| --- | --- | --- | --- | --- | --- | --- | --- |
|  | Adult Male | | | | | | |
|  | FDG | RGD | NOC | OC | ENC15 | ENC18 |  |
| Adrenals | 9.39E-03 | 7.60E-03 | 1.09E-02 | 1.15E-02 | 2.23E-02 | 3.36E-02 |  |
| Brain | 5.73E-03 | 1.47E-03 | 1.35E-03 | 1.07E-03 | 7.59E-04 | 8.53E-04 |  |
| Breasts | 5.93E-03 | 4.25E-03 | 3.84E-03 | 3.56E-03 | 2.94E-03 | 3.69E-03 |  |
| Gallbladder Wall | 1.04E-02 | 8.74E-03 | 1.05E-02 | 1.04E-02 | 1.49E-02 | 2.06E-02 ± |  |
| LLI Wall | 1.92E-01 ‡ | 1.50E-01 ‡ | 2.70E-01 ‡ | 1.97E-01 ‡ | 9.91E-02 § | 8.51E-02 § |  |
| Small Intestine | 1.91E-02 | 1.73E-02 | 2.11E-02 | 1.92E-02 | 1.75E-02 | 1.76E-02 |  |
| Stomach Wall | 9.73E-03 | 7.65E-03 | 8.79E-03 | 8.37E-03 | 1.05E-02 | 1.42E-02 |  |
| ULI Wall | 1.49E-02 | 1.35E-02 | 1.54E-02 | 1.45E-02 | 1.45E-02 | 1.54E-02 |  |
| Heart Wall | 2.60E-02 | 6.02E-03 | 8.25E-03 | 7.09E-03 | 5.93E-03 | 7.47E-03 |  |
| Kidneys | 3.58E-02 § | 4.41E-02 § | 1.68E-01 § | 2.06E-01 § | 6.14E-01 ‡ | 9.67E-01† |  |
| Liver | 9.19E-03 | 8.14E-03 | 1.06E-02 | 9.72E-03 | 1.06E-02 | 1.51E-02 |  |
| Lungs | 6.86E-03 | 3.96E-03 | 4.86E-03 | 4.36E-03 | 4.04E-03 | 5.36E-03 |  |
| Muscle | 1.15E-02 | 1.06E-02 | 1.11E-02 | 1.07E-02 | 1.00E-02 | 9.79E-03 |  |
| Testes* | 1.82E-02 | 1.89E-02 | 1.92E-02 | 1.89E-02 | 1.53E-02 | 9.78E-03 |  |
| Pancreas | 9.88E-03 | 7.83E-03 | 9.96E-03 | 1.02E-02 | 1.70E-02 | 2.48E-02 |  |
| Red Marrow | 1.08E-02 | 9.28E-03 | 1.08E-02 | 1.02E-02 | 1.08E-02 | 1.26E-02 |  |
| Osteogenic Cells | 1.26E-02 | 1.00E-02 | 9.79E-03 | 9.13E-03 | 8.08E-03 | 9.17E-03 |  |
| Skin | 6.97E-03 | 5.85E-03 | 5.76E-03 | 5.49E-03 | 4.98E-03 | 5.30E-03 |  |
| Spleen | 9.14E-03 | 7.40E-03 | 1.04E-02 | 1.09E-02 | 2.07E-02 | 3.08E-02 |  |
| Thymus | 7.56E-03 | 5.18E-03 | 4.65E-03 | 4.28E-03 | 3.37E-03 | 4.18E-03 |  |
| Thyroid | 6.91E-03 | 5.01E-03 | 4.24E-03 | 3.89E-03 | 2.77E-03 | 3.34E-03 |  |
| Urinary Bladder Wall | 5.53E-01 † | 6.89E-01 † | 7.05E-01 † | 7.31E-01 † | 6.31E-01 † | 3.20E-01 ±‡ |  |
| Uterus | N/A | N/A | N/A | N/A | N/A | N/A |  |
| Total Body | 1.19E-02 | 1.06E-02 | 1.19E-02 | 1.15E-02 | 1.23E-02 | 1.36E-02 |  |
| Effective Dose (mSv/MBq), Average ± SD | 6.24E-02 | 6.30E-02 | 8.07E-02 | 7.23E-02 | 6.09E-02 | 5.97E-02 |  |

**Supplementary table 10.** Predicted clinical absorbed organ doses and whole-body effective doses of PET radiotracers using non-normalised (preclinical) adult female source organ residence times from iterative reconstructions. Legend: *: Ovaries data from female phantoms; †: Absorbed organ dose with highest upper bound of 95% confidence interval for dosimetry model. ‡: Absorbed organ dose with second highest upper bound of 95% confidence interval for dosimetry model; §: Absorbed organ dose with third highest upper bound of 95% confidence interval for dosimetry model.

| Target Organ | Estimated Absorbed Dose of Organ (mGy/MBq), Average ± SD | | | | | | |
| --- | --- | --- | --- | --- | --- | --- | --- |
|  | Adult Female | | | | | | |
|  | FDG | RGD | NOC | OC | ENC15 | ENC18 |  |
| Adrenals | 1.20E-02 | 9.77E-03 | 1.42E-02 | 1.51E-02 | 2.97E-02 | 4.47E-02 |  |
| Brain | 6.80E-03 | 1.87E-03 | 1.70E-03 | 1.37E-03 | 9.77E-04 | 1.11E-03 |  |
| Breasts | 7.63E-03 | 5.49E-03 | 4.98E-03 | 4.61E-03 | 3.78E-03 | 4.67E-03 |  |
| Gallbladder Wall | 1.27E-02 | 1.07E-02 | 1.26E-02 | 1.24E-02 | 1.67E-02 | 2.26E-02 |  |
| LLI Wall | 2.13E-01 ‡ | 1.68E-01 ‡ | 2.98E-01 ‡ | 2.19E-01 ‡ | 1.12E-01§ | 9.51E-02 § |  |
| Small Intestine | 2.39E-02 ± | 2.22E-02 | 2.72E-02 | 2.48E-02 | 2.29E-02 | 2.26E-02 |  |
| Stomach Wall | 1.21E-02 | 9.44E-03 | 1.07E-02 | 1.01E-02 | 1.20E-02 | 1.60E-02 |  |
| ULI Wall | 1.93E-02 | 1.78E-02 | 2.02E-02 | 1.91E-02 | 1.87E-02 | 1.94E-02 |  |
| Heart Wall | 3.36E-02 | 7.79E-03 | 1.07E-02 | 9.20E-03 | 7.67E-03 | 9.61E-03 |  |
| Kidneys | 3.95E-02 | 4.85E-02§ | 1.83E-01 § | 2.23E-01 § | 6.65E-01 ‡ | 1.05E+00 † |  |
| Liver | 1.20E-02 | 1.06E-02 | 1.37E-02 | 1.24E-02 | 1.30E-02 | 1.84E-02 |  |
| Lungs | 8.85E-03 | 5.19E-03 | 6.39E-03 | 5.78E-03 | 5.54E-03 | 7.32E-03 |  |
| Muscle | 1.42E-02 | 1.30E-02 | 1.36E-02 | 1.32E-02 | 1.24E-02 | 1.21E-02 |  |
| Ovaries* | 4.21E-02 | 4.12E-02 | 5.03E-02 | 4.53E-02 | 3.46E-02 | 2.53E-02 |  |
| Pancreas | 1.23E-02 | 9.72E-03 | 1.22E-02 | 1.23E-02 | 1.99E-02 | 2.88E-02 |  |
| Red Marrow | 1.35E-02 | 1.18E-02 | 1.36E-02 | 1.28E-02 | 1.34E-02 | 1.51E-02 |  |
| Osteogenic Cells | 1.64E-02 | 1.30E-02 | 1.27E-02 | 1.19E-02 | 1.06E-02 | 1.22E-02 |  |
| Skin | 8.59E-03 | 7.18E-03 | 7.03E-03 | 6.69E-03 | 5.99E-03 | 6.35E-03 |  |
| Spleen | 1.16E-02 | 9.42E-03 | 1.30E-02 | 1.36E-02 | 2.55E-02 | 3.78E-02 |  |
| Thymus | 9.63E-03 | 6.68E-03 | 5.97E-03 | 5.49E-03 | 4.28E-03 | 5.27E-03 |  |
| Thyroid | 8.07E-03 | 5.90E-03 | 4.98E-03 | 4.57E-03 | 3.19E-03 | 3.83E-03 |  |
| Urinary Bladder Wall | 7.75E-01 † | 9.66E-01 † | 9.90E-01 † | 1.02E+00 † | 8.86E-01 † | 4.48E-01 ‡ |  |
| Uterus | 5.40E-02 § | 6.04E-02 | 6.40E-02 | 6.36E-02 | 5.36E-02 | 3.26E-02 |  |
| Total Body | 1.49E-02 | 1.33E-02 | 1.50E-02 | 1.44E-02 | 1.55E-02 | 1.72E-02 |  |
| Effective Dose (mSv/MBq), Average ± SD | 7.95E-02 | 8.22E-02 | 1.02E-01 | 9.32E-02 | 7.86E-02 | 7.18E-02 |  |

**Supplementary table 11.** Predicted clinical absorbed organ doses and whole-body effective doses of PET radiotracers using normalised adult male source organ residence times from iterative reconstructions. Legend: *: Testes data from male phantoms; †: Absorbed organ dose with highest upper bound of 95% confidence interval for dosimetry model; ‡: Absorbed organ dose with second highest upper bound of 95% confidence interval for dosimetry model; §: Absorbed organ dose with third highest upper bound of 95% confidence interval for dosimetry model.

| Target Organ | Estimated Absorbed Dose of Organ (mGy/MBq), Average | | | | | |
| --- | --- | --- | --- | --- | --- | --- |
|  | Adult Male | | | | | |
|  | FDG | RGD | NOC | OC | ENC15 | ENC18 |
| Adrenals | 9.55E-03 | 6.76E-03 | 8.55E-03 | 8.69E-03 | 1.45E-02 | 2.16E-02 |
| Brain | 1.47E-02 | 2.27E-03 | 2.27E-03 | 1.58E-03 | 1.15E-03 | 1.21E-03 |
| Breasts | 6.86E-03 | 4.34E-03 | 3.99E-03 | 3.63E-03 | 2.71E-03 | 3.34E-03 |
| Gallbladder Wall | 9.38E-03 | 7.11E-03 | 7.85E-03 | 7.58E-03 | 9.64E-03 | 1.36E-02 |
| LLI Wall | 7.18E-02 § | 5.51E-02 § | 9.71E-02 § | 7.08E-02 § | 3.57E-02 § | 3.22E-02 § |
| Small Intestine | 1.15E-02 | 9.30E-03 | 1.03E-02 | 9.38E-03 | 8.79E-03 | 1.03E-02 |
| Stomach Wall | 9.21E-03 | 6.44E-03 | 6.72E-03 | 6.33E-03 | 7.11E-03 | 9.72E-03 |
| ULI Wall | 1.01E-02 | 8.07E-03 | 8.43E-03 | 7.88E-03 | 7.82E-03 | 9.55E-03 |
| Heart Wall | 8.18E-02 ‡ | 1.28E-02 | 2.04E-02 | 1.66E-02 | 1.06E-02 | 1.19E-02 |
| Kidneys | 2.30E-02 | 2.74E-02 ‡ | 1.02E-01 ‡ | 1.25E-01 ‡ | 3.71E-01 † | 5.86E-01 † |
| Liver | 9.11E-03 | 7.32E-03 | 9.17E-03 | 8.11E-03 | 7.15E-03 | 1.02E-02 |
| Lungs | 1.46E-02 | 6.59E-03 | 9.31E-03 | 7.86E-03 | 5.23E-03 | 6.29E-03 |
| Muscle | 8.37E-03 | 6.45E-03 | 6.33E-03 | 5.95E-03 | 5.39E-03 | 6.22E-03 |
| Testes* | 9.57E-03 | 8.41E-03 | 8.00E-03 | 7.60E-03 | 5.77E-03 | 4.79E-03 |
| Pancreas | 1.00E-02 | 6.93E-03 | 7.96E-03 | 7.85E-03 | 1.13E-02 | 1.64E-02 |
| Red Marrow | 8.31E-03 | 6.11E-03 | 6.49E-03 | 6.06E-03 | 6.33E-03 | 8.08E-03 |
| Osteogenic Cells | 1.16E-02 | 8.36E-03 | 7.63E-03 | 7.04E-03 | 5.78E-03 | 7.04E-03 |
| Skin | 5.99E-03 | 4.44E-03 | 4.09E-03 | 3.81E-03 | 3.21E-03 | 3.84E-03 |
| Spleen | 8.72E-03 | 6.39E-03 | 7.87E-03 | 8.00E-03 | 1.34E-02 | 1.99E-02 |
| Thymus | 9.60E-03 | 5.44E-03 | 5.08E-03 | 4.58E-03 | 3.29E-03 | 4.00E-03 |
| Thyroid | 7.39E-03 | 5.10E-03 | 4.34E-03 | 3.96E-03 | 2.74E-03 | 3.27E-03 |
| Urinary Bladder Wall | 1.43E-01 † | 1.75E-01 † | 1.79E-01† | 1.85E-01 † | 1.59E-01 ‡ | 8.22E-02 ‡ |
| Uterus | N/A | N/A | N/A | N/A | N/A | N/A |
| Total Body | 9.30E-03 | 6.69E-03 | 7.07E-03 | 6.66E-03 | 6.92E-03 | 8.66E-03 |
| Effective Dose (mSv/MBq), Average ± SD | 2.46E-02 | 2.17E-02 | 2.86E-02 | 2.60E-02 | 2.66E-02 | 2.85E-02 |

**Supplementary table 12.** Predicted clinical absorbed organ doses and whole-body effective doses of PET radiotracers using normalised adult female source organ residence times from iterative reconstructions. Legend: *: Ovaries data from female phantoms; †: Absorbed organ dose with highest upper bound of 95% confidence interval for dosimetry model; ‡: Absorbed organ dose with second highest upper bound of 95% confidence interval for dosimetry model; §: Absorbed organ dose with third highest upper bound of 95% confidence interval for dosimetry model.

| Target Organ | Estimated Absorbed Dose of Organ (mGy/MBq), Average | | | | | |
| --- | --- | --- | --- | --- | --- | --- |
|  | Adult female | | | | | |
| Adrenals | FDG | RGD | NOC | OC | ENC15 | ENC18 |
| Brain | 1.19E-02 | 8.66E-03 | 1.13E-02 | 1.16E-02 | 2.05E-02 | 3.07E-02 |
| Breasts | 1.84E-02 | 2.91E-03 | 2.89E-03 | 2.02E-03 | 1.48E-03 | 1.56E-03 |
| Gallbladder Wall | 8.62E-03 | 5.51E-03 | 5.05E-03 | 4.60E-03 | 3.46E-03 | 4.27E-03 |
| LLI Wall | 1.11E-02 | 8.45E-03 | 9.32E-03 | 8.97E-03 | 1.13E-02 | 1.58E-02 |
| Small Intestine | 8.33E-02 § | 6.42E-02 ‡ | 1.12E-01 § | 8.20E-02 § | 4.18E-02 § | 3.77E-02 § |
| Stomach Wall | 1.38E-02 | 1.13E-02 | 1.29E-02 | 1.18E-02 | 1.14E-02 | 1.35E-02 |
| ULI Wall | 1.15E-02 | 8.02E-03 | 8.33E-03 | 7.82E-03 | 8.58E-03 | 1.17E-02 |
| Heart Wall | 1.28E-02 | 1.03E-02 | 1.09E-02 | 1.02E-02 | 1.02E-02 | 1.24E-02 |
| Kidneys | 9.59E-02 ‡ | 1.53E-02 | 2.42E-02 | 1.97E-02 | 1.28E-02 | 1.45E-02 |
| Liver | 2.72E-02 | 3.23E-02 § | 1.20E-01 ‡ | 1.46E-01 ‡ | 4.36E-01 † | 6.87E-01 † |
| Lungs | 1.15E-02 | 9.20E-03 | 1.15E-02 | 1.02E-02 | 9.15E-03 | 1.31E-02 |
| Muscle | 1.79E-02 | 8.21E-03 | 1.16E-02 | 9.82E-03 | 6.82E-03 | 8.33E-03 |
| Ovaries * | 1.03E-02 | 7.96E-03 | 7.85E-03 | 7.37E-03 | 6.79E-03 ± | 7.94E-03 |
| Pancreas | 1.95E-02 | 1.68E-02 | 1.96E-02 | 1.73E-02 | 1.31E-02 | 1.20E-02 ± |
| Red Marrow | 1.23E-02 | 8.65E-03 | 9.91E-03 | 9.74E-03 | 1.40E-02 | 2.04E-02 |
| Osteogenic Cells | 1.02E-02 | 7.60E-03 | 8.08E-03 | 7.56E-03 | 7.95E-03 | 1.01E-02 |
| Skin | 1.51E-02 | 1.09E-02 | 1.00E-02 | 9.25E-03 | 7.76E-03 | 9.55E-03 |
| Spleen | 7.40E-03 | 5.48E-03 | 5.05E-03 | 4.70E-03 | 3.97E-03 | 4.75E-03 |
| Thymus | 1.10E-02 | 8.11E-03 | 1.01E-02 | 1.03E-02 | 1.75E-02 | 2.60E-02 |
| Thyroid | 1.16E-02 | 6.90E-03 | 6.36E-03 | 5.75E-03 | 4.16E-03 | 5.06E-03 |
| Urinary Bladder Wall | 8.48E-03 | 5.97E-03 | 5.08E-03 | 4.63E-03 | 3.18E-03 | 3.79E-03 |
| Uterus | 1.93E-01 † | 2.38E-01 † | 2.43E-01 † | 2.51E-01 † | 2.16E-01 ‡ | 1.11E-01 ‡ |
| Total Body | 2.12E-02 | 2.06E-02 | 2.12E-02 | 2.05E-02 | 1.69E-02 | 1.28E-02 |
| Effective Dose (mSv/MBq), Average ± SD | 1.15E-02 | 8.33E-03 | 8.89E-03 | 8.39E-03 | 8.98E-03 | 1.14E-02 |

**Supplementary figure 4.** Linear Regression for Scatterplots of Predicted Clinical Absorbed Organ Doses of Select PET Radiotracers as a Function of True Clinical Absorbed Doses of the Radiotracers (Including Analysis of Absorbed Doses of the Lower Large Intestinal Wall). Linear regressions plotted for various predictive dosimetry models of **(a)** 2-deoxy-2-[^18^F]fluoro-d-glucose and **(b)** [^18^F]AlF-NOTA-octreotide. Error bars = Average ± SEM.


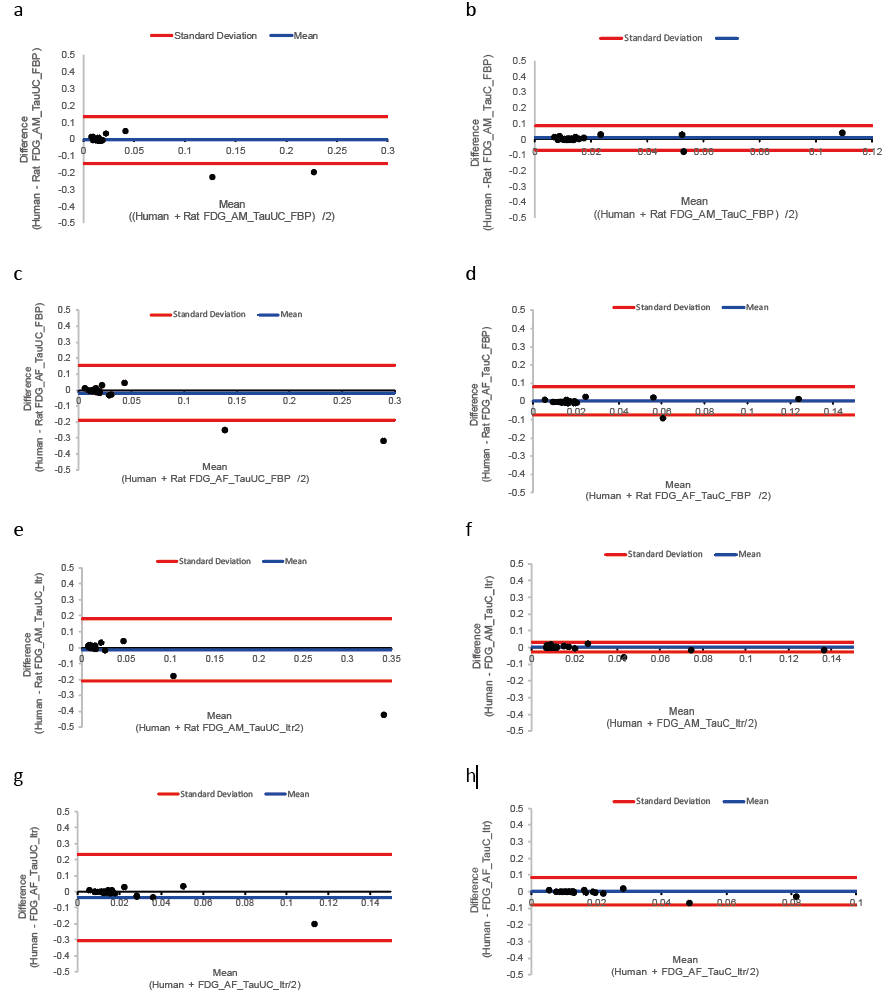


**Supplementary figure 5**. Bland-Altman plots assessing agreement between predicted clinical absorbed organ doses predicted from preclinical data for [^18^F]FDG in all tissues including LLI. **(a)** Human predicted dose and FDG adult male TauUC FBP, **(b)** Human predicted dose and FDG rat adult male TauC FBP, **(c)** Human predicted dose and FDG rat adult female TauUC FBP, **(d)** Human predicted dose and FDG rat adult female TauUC FBP, **(e)** Human predicted dose and FDG rat adult male TauUC FBP Itr, **(f)** Human predicted dose and FDG rat adult male TauC FBP Itr **(g)** Human predicted dose and FDG rat adult female TauUC FBP Itr and **(h)** Human predicted dose and OC rat adult female TauC FBP Itr.


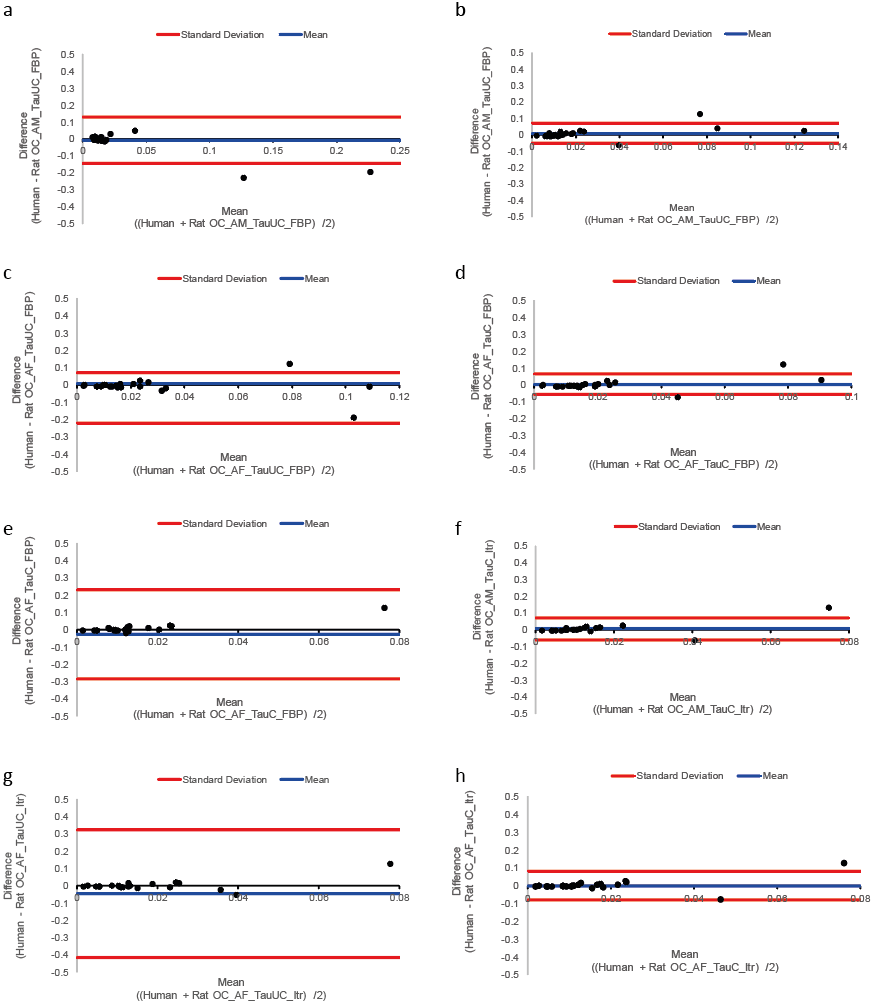


**Supplementary figure 6**. Bland-Altman plots assessing agreement between predicted clinical absorbed organ doses predicted from preclinical data for [^18^F]AlF-NOTA-NOC in all tissues including LLI. **(a)** Human predicted dose and OC adult male TauUC FBP, **(b)** Human predicted dose and OC rat adult male TauC FBP, **(c)** Human predicted dose and OC rat adult female TauUC FBP, **(d)** Human predicted dose and OC rat adult female TauUC FBP, **(e)** Human predicted dose and OC rat adult male TauUC FBP Itr, **(f)** Human predicted dose and OC rat adult male TauC FBP Itr **(g)** Human predicted dose and FDG rat adult female TauUC FBP Itr and **(h)** Human predicted dose and OC rat adult female TauC FBP Itr.

**References**

1. Valentin, J. & Streffer, C. Basic anatomical and physiological data for use in radiological protection: Reference values - ICRP Publication 89. *Ann. ICRP* **32**, 1–277 (2002).

2. FLS, T. Nonclinical pharmacokinetic studies. in *Pharmacokinetics* (eds. Welling, P. & Tse, F.) (Marcel Dekker, 1998).

3. Spector, W. S. & (U.S.), N. R. C. *Handbook of biological data ; prepared under the direction of the Committee on the Handbook of Biological Data, Division of Biology and Agriculture, the National Academy of Sciences the National Resear.* (Saunders, 1961).

4. Stabin, M. G., Peterson, T. E., Holburn, G. E. & Emmons, M. A. Voxel-based mouse and rat models for internal dose calculations. *J. Nucl. Med.* **47**, 655–659 (2006).
